# Single nucleotide variants in the IL33 and IL1RL1 (ST2) genes are associated with periodontitis and with *Aggregatibacter actinomycetemcomitans* in the dental plaque biofilm: A putative role in understanding the host immune response in periodontitis

**DOI:** 10.1371/journal.pone.0283179

**Published:** 2023-03-22

**Authors:** Soraya C. Trindade, Mabel P. P. Lopes, Tatiane T. M. C. Oliveira, Milca J. Silva, Gerson A. Queiroz, Talita S. Jesus, Ellen K. N. Santos, Paulo C. Carvalho-Filho, Michelle M. L. Falcão, Patrícia M. Miranda, Rebeca P. B. Santos, Camila A. Figueiredo, Álvaro A. Cruz, Gregory J. Seymour, Isaac S. Gomes-Filho

**Affiliations:** 1 Postgraduate Program in Immunology, Federal University of Bahia, Salvador, Bahia, Brazil; 2 Department of Health, Feira de Santana State University, Feira de Santana, Bahia, Brazil; 3 Postgraduate Program in Health Sciences, Federal University of Bahia, Salvador, Bahia, Brazil; 4 Oral Health Centre, The University of Queensland, Herston, Queensland, Australia; Universidade de Trás-os-Montes e Alto Douro: Universidade de Tras-os-Montes e Alto Douro, PORTUGAL

## Abstract

The Interleukin (IL)-33 is important in several inflammatory diseases and its cellular receptor is the Interleukin 1 receptor-like 1 (IL1RL1), also called suppression of tumorigenicity 2 ligand (ST2L). This study investigated associations between single nucleotide variants (SNVs) in the IL33 gene and in the IL1RL1 (ST2) gene with periodontitis. Additionally, aimed to determine the role of *Aggregatibacter actinomycetemcomitans* (*Aa*) relative amount in the subgingival biofilm in these associations. A cross-sectional study was carried out with 506 individuals that answered a structured questionnaire used to collect their health status, socioeconomic-demographic, and behavioral characteristics. Periodontal examination was performed to determine the presence and severity of periodontitis, and subgingival biofilm samples were collected to quantify the relative amount of *Aa* by real time polymerase chain reaction. Human genomic DNA was extracted from whole blood cells and SNV genotyping was performed. Logistic regression estimated the association measurements, odds ratio (OR), and 95% confidence interval (95%CI), between the IL33 and ST2 genes with periodontitis, and subgroup analyses assessed the relative amount of *Aa* in these associations. 23% of individuals had periodontitis. Adjusted measurements showed a statistically significant inverse association between two SNVs of the ST2; rs148548829 (C allele) and rs10206753 (G allele). These two alleles together with a third SNV, the rs11693204 (A allele), were inversely associated with moderate periodontitis. One SNV of the IL33 gene also showed a statistically significant inverse association with moderate periodontitis. Nine SNVs of the ST2 gene were inversely associated with the relative amount of *Aa*. In the high *Aa* subgroup, there was a direct association between 11 SNVs of the ST2 gene and moderate periodontitis and two SNVs of the ST2 gene and severe periodontitis, and eight SNVs of the ST2 gene and periodontitis. These exploratory findings of genetic variants in IL-33/ST2 axis support the concept that the different tissue responses among individuals with periodontitis may be modulated by the host’s genetics, influencing the physiopathology of the disease.

## Introduction

Periodontitis is a frequently occurring chronic disease worldwide, which is initiated and maintained by the presence of a dysbiotic subgingival microbial biofilm. Genetic and environmental factors, as well as lifestyle, play a relevant role in disease development [1, 2]. Each individual diagnosed with periodontitis can present with a different tissue response that may be influenced by the host’s genetics. Evidence indicates that some genes play a role not only in predisposition to periodontitis but also in disease progression [2–5].

The investigation of genetic factors in periodontitis has evolved over the years [6–8] and more recently genome-wide association studies (GWAS) have contributed to the identification of several candidate genes [9–16], which may contribute to an understanding of how genetic factors may influence the physiopathology of this disease.

Despite the identification of several candidate genes, so far, GWAS have not confirmed any direct associations between genetic signatures and periodontitis or its surrogate markers [9–16]. Among candidate genes, some of such studies have suggested the influence of the Interleukin-33 gene in the pathogenesis of periodontitis [10, 12, 16].

Interleukin (IL)-33 is a member of the IL-1 family of cytokines, known to be important in several inflammatory diseases [17]. In the pathogenesis of periodontitis, the cellular release of IL-33 can exert a damage-associated molecular pattern-like (DAMP) function [18]. Furthermore, this cytokine can activate cells expressing the IL-33 receptor, the Interleukin 1 receptor-like 1 (IL1RL1), also called suppression of tumorigenicity 2 ligand (ST2L), producing an inflammatory environment [17–22].

Additionally, IL-33 may participate in the bone resorption process observed in periodontitis in two ways: by disrupting the RANK (receptor activator of nuclear-kB factor) / RANKL (RANK ligand) / OPG (osteoprotegerin) axis [23, 24] and by increasing the expression of osteoclast precursor transcription factors, favoring the differentiation and activation of these cells [25].

Both the IL-33 gene and IL1RL1 gene (ST2 gene) have been implicated in the immune response in periodontitis, as well as in chronic disease of the airways, such as asthma [26–29]. However, studies attempting to evaluate associations between these genes and periodontitis are lacking. Equally, factors that may interfere in this relationship, such as the presence of the pathogenic bacterium, *Aggregatibacter actinomycetemcomitans*, have not been studied.

Accordingly, the present study aimed to investigate associations between single nucleotide variants (SNVs) in the IL-33 gene and in the ST2 gene with periodontitis and between these SNVs and the relative amount of *A*. *actinomycetemcomitans* in the subgingival biofilm.

## Materials and methods

### Population and ethical considerations

A cross-sectional study was conducted involving individuals who sought care at a public health service clinic in Salvador, Bahia, Brazil, from January 2013 to November 2014. It was a black / mixed-race population with African, indigenous, and European ancestry. This study was approved by the institutional review board of the Feira de Santana State University, Bahia, Brazil (protocol no. 43131615.3.0000.0053), and was conducted in accordance with the Helsinki Declaration of 1975, as revised in 2013. All participants signed informed consent forms.

### Eligibility criteria

The study sample included individuals who were seen at the abovementioned public health service. Individuals under the age of 18, pregnant women, relatives, and spouses of included individuals, or persons who had undergone periodontal treatment in the six months prior to their visit, were not included.

### Sample size

The sample size was calculated considering a frequency of 16% of individuals with periodontitis (outcome) among those not exposed to the variant rs23811416. The power was estimated at 99%, with a 95% confidence level and 3:1 ratio between individuals with periodontitis and without periodontitis. Thus, the estimated minimum sample size was 117 individuals in the periodontitis group (individuals with a confirmed diagnosis of periodontitis) and 350 in the group without periodontitis.

### Data collection

A structured questionnaire was applied through interviews to obtain socioeconomic-demographic data, information on lifestyle and health habits, the presence of comorbidities, and access to dental care.

### Dependent variables

#### Periodontitis

A single trained periodontist evaluated the following periodontal parameters at six sites of each tooth, excluding the third molars: recession measurement, probing depth, clinical attachment level, and bleeding on probing [30]. Intra-examiner reliability of recession and probing depth measurements was assessed using the Bland e Altman method (0,067 and 0,071, respectively) in 10% of the sample.

The diagnosis of periodontitis included the presence of at least four teeth with one or more sites with a probing depth (PD) ≥ 4mm, clinical attachment level (CAL) ≥ 3mm and bleeding on probing (BOP) at the same site [30]. Participants were classified in two groups according to the presence or absence of periodontitis, as well as the severity of the disease [30]. Severe periodontitis included the presence of at least four teeth with one or more sites with PD ≥ 5mm, CAL ≥ 5mm, and BOP at the same site. Moderate periodontitis included the presence of at least four teeth with one or more sites with PD ≥ 4mm, CAL ≥ 3m,m and BOP at the same site [30].

*Aggregatibacter actinomycetemcomitans* relative amount. Following periodontal examination and supragingival biofilm removal, the subgingival biofilm was collected using a periodontal curette (Hu-Friedy, Chicago, IL, USA). The site with the greatest probing depth of each sextant was selected for sample collection. Subgingival biofilm samples were placed in a sterile saline-phosphate buffer.

Bacterial DNA from the subgingival biofilm samples was extracted using a Genomic DNA kit (PureLink™ Genomic DNA Mini Kit, Invitrogen, Carlsbad, CA, USA) in accordance with the manufacturer´s instructions.

The relative quantification of *A*. *actinomycetencomitans* was performed using a quantitative polymerase chain reaction (qPCR) assay (TaqMan^®^ qPCR Assay, Applied Biosystems, Foster City, CA, USA). The probe sequence was: 5’ - 6—FAM—CRA ACA GGA TTA GAT ACC CTG GTA GTC CRC—BHQ 1–3’. Specific primer sequences were forward—5’- GAT GTA CTG ACG CTG ATG TGC -3’ and reverse—5’- CCC AAA TCG ACA CCG TTT ACA G -3’ [31].

The PCR mix (final volume of 12.5 μL) for each reaction consisted of: 10X buffer (1.25 μL) containing 50 mM MgCl2 (0.38 μL), 4x2.5 mM dNTPs (1 μL), 10 μM of forward primer (0.38 μL), 10 μM of reverse primer (0.38 μL), Taq 5U/μL (0.05 μL), RNAse free water (6.33 μL), 10μM of probe sequence (0.25 μL) and DNA (2.5 μL). qPCR cycle conditions were as follows: an initial denaturation cycle at 94°C for 1 min, followed by 45 cycles at 94°C for 20 sec and a final annealing step at 58°C for 35 sec.

Participants were classified in two groups according to the relative quantification of *A*. *actinomycetemcomitans* in the biofilm, dichotomized according to the median value (20.13%), comparing groups with and without periodontitis [31].

### Independent variables

#### Single nucleotide variants (SNVs) of the IL33 and ST2 genes

After genotyping, 14 IL33 SNVs and 47 ST2 SNVs were selected according to their biological function, location, minor allele frequency, and their prior associations with clinical conditions that related to IL-33 and ST2 gene expression and inflammatory diseases, using the National Center for Biotechnology Information (NCBI) online database [32]. Ensembl (https://www.ensembl.org/index.html) and Regulome Db (https://regulomedb.org/) were used for SNP annotation. This prospecting information is arranged in S1 Table.

For the genotyping, peripheral blood samples of all participants were collected by venipuncture at the antecubital fossa into EDTA-containing tubes and DNA was extracted using a DNA extraction kit (Gentra Puregene Blood Kit, Qiagen, Hilden, Germany) in accordance with the manufacturer´s instructions.

The genotyping was performed using a Multi-Ethnic Global Array (MEGA) platform (Infinium® Multi-Ethnic Global Array—MEGA, Illumina, San Diego, CA, USA). After genotyping, SNVs were extracted from the interest region of the IL33 and ST2 genes.

Ancestry inference was performed using principal component (PC) analysis. For this, PC1 and PC2 axes were calculated. The following filters were applied for quality control: genotyping error rate ≤ 10%; Hardy-Weinberg equilibrium (HWE) >0.05; and minor allele frequency (MAF) < 1%. The Imputation strategy had been performed in the Michigan Imputation Server [33]. The reference panel was the African American Panel GRCh37/hg19 of the Consortium on Asthma among African-ancestry Populations in the Americas (CAAPA) [34].

For the present analysis, the quality controls before were non-biallelic SNVs; HWE (< 1x10^-4^); and MAF (< 1x10^-4^). The parameters were: rsq filter of 0.1; phasing Eagle v2.4; and population mixed. The quality controls after were average calls > 0.95; r2 > 0.80; and minor allele count (MAC) > 10.

### Statistical analysis

Data analysis was performed using SPSS version 21 and Plink version 1.07. Bivariate analysis was used to compare groups with and without periodontitis in relation to socioeconomic-demographic data, lifestyle, health habits, presence of comorbidities and access to dental care, using the Chi-square or Fisher’s Exact tests. The statistical significance level used was p ≤ 0.05.

To estimate the association between the single nucleotide variants (SNVs) of the IL33 and ST2 genes with periodontitis, with its moderate and severe levels of severity and with the *A*. *actinomycetemcomitans* relative amount, eight standard analysis models were performed for each of the 14 IL33 SNVs and for each of the 47 ST2 SNVs. The models were, as follows: 1. SNVs of the IL33 gene and periodontitis; 2. SNVs of the ST2 gene and periodontitis; 3. SNVs of the IL33 gene and severe periodontitis; 4. SNVs of the ST2 gene and severe periodontitis; 5. SNVs of the IL33 gene and moderate periodontitis; 6. SNVs of the ST2 gene and moderate periodontitis; 7. SNVs of the IL33 gene and *A*. *actinomycetemcomitans* relative amount; 8. SNVs of the ST2 gene and *A*. *actinomycetemcomitans* relative amount.

Thereby, to obtain the mentioned association measurements, crude odds ratios (OR) and 95% confidence intervals were estimated using the Mantel-Haenszel test.

The presence of effect modifiers and confounders was investigated through the construction of a conceptual theoretical model on the investigated associations. Then, the presence of interaction covariables was also investigated using the maximum likelihood test (p < 0.05) and, in sequence, the identification of confounding covariables was performed using the backward strategy if the covariable had produced a relative difference of 10% in the association measurement in relation to the saturated model.

In the multiple logistic regression analysis, the adjusted association measurements were estimated considering the dominant genetic model. Therefore, the following covariables were selected as confounders: total years of education, diagnosis of asthma, flossing, age, body mass index (BMI), mouth breathing and PC1. Depending on the model, the number of confounders was altered. Interaction analysis did not identify presence of effect modifiers.

Even though, to explore the influence of the gene environment interaction on the association between the SNVs of the IL33 and ST2 genes and periodontitis, a subgroup analysis was performed using the variable *A*. *actinomycetemcomitans* relative amount, dividing the association models in groups with high and low quantification of this bacterium in the biofilm.

In all analyzes, to correct the false-positive rate, a permutation procedure was performed to obtain a permutation p-value based on the number of repetitions performed in the multiple analysis (p ≤ 0.05).

### *In silico* analysis

Characterization and information regarding the function of each variant was verified on NCBI database and the regulatory potential activity was verified on RegulomeDB [35], which attributes a score of 1 to 7 related to the probability of the impact of the SNV on gene expression. The lower the attributable score, the greater is the chance to affect binding; score 7 means that no information is available in this regard.

An expression quantitative trait locus (eQTL) analysis was done using the Genotype-Tissue Expression (GTEx) Portal [36], to predict the gene expression profile in whole blood tissue. In addition, a linkage disequilibrium (LD) analysis, as well as the degree of confidence in r^2^ value, were performed using Haploview 4.2.

## Results

The study sample consisted of 506 individuals: 23% (117) with periodontitis, and 77% (389) without the disease. The frequencies of moderate and severe periodontitis were 7.5% and 15.2%, respectively. Table 1 showed the socioeconomic-demographic characteristics, related to lifestyle, general and oral health of the sample. Statistically significant differences in the following covariables were seen between individuals with and without periodontitis: age, total years of education, family income, mouth-breathing habit, flossing, a diagnosis of hypertension, a diagnosis of cardiovascular disease, body mass index (BMI) and a diagnosis of asthma.

**Table 1:** Distribution of socioeconomic-demographic characteristics of the sample according to the presence of periodontitis, related to lifestyle, general health, and oral health.

Characteristics	Without Periodontits	With Periodontits	p
	N = 389	N = 117	
	N (%)	N (%)	
**Age (years)**			
>39	253 (65%)	88 (35.2%)	0.04
**Gender**			
Male	62 (15.9%)	23 (19.7%)	0.34
**Race / Skin Color**			
Black/mixed-race	328 (84.3%)	93 (74.5%)	0.22
**Total Years of Education**			
≤ 4 years	325 (83.5%)	80 (68.4%)	0.00
**Family Income (Monthly Minimum Wage)**			
≤ 1 monthly minimum wage (R$724.00/US$ 304.2 in 2014)	87 (28.4%)	41 (35%)	0.00
**Current Smoking Habit**			
Yes	15 (3.9%)	2 (1.7%)	0.38
**Regular Physical Activity**			
No	260 (66.8%)	74 (63.2%)	0.47
**Frequency Of Dental Visits**			
Never or ≥ 1 year	204 (52.4%)	76 (65%)	0.16
**Received Guidance Regarding Oral Hygiene**			
No	79 (20.3%)	26 (22.2%)	0.65
**Mouth-breathing Habit**			
Yes	228 (58.6%)	91 (77.8%)	0.00
**Frequency of Tooth Brushing**			
<3x per day	160 (41.1%)	53 (45.3%)	0.42
**Flossing**			
No	168 (43.2%)	72 (61.5%)	0.00
**Diagnosis of Hypertension**			
Yes	99 (25.4%)	44 (36.8%)	<0.01
**Diagnosis of Diabetes**			
Yes	18 (4.6%)	9 (7.7%)	0.14
**Diagnosis of Cardiovascular Disease**			
Yes	10 (2.6%)	8 (6.8%)	0.03
**Diagnosis of Asthma**			
Severe asthma	256 (66%)	34 (29.1%)	<0.00
**Body Mass Index—BMI (weight/height^2^)**			
≥25 kg/m^2^	265 (68.1%)	91 (77.8%)	0.04

N: total number of individuals; p: statistical significance level (p≤0.05).

Table 2 shows statistically significant adjusted association measurements between the SNVs of the IL33 gene and periodontitis, as well as the SNVs of the ST2 gene and periodontitis, considering the severe and moderate severity levels, using dominant genetic model. The other non-statistically significant association measurements are in S2 and S3 Tables.

**Table 2:** Statistically significant adjusted association measurements, odds ratio and 95% confidence interval, between the SNVs of the IL-33 gene and periodontitis, as well as the SNVs of the ST2 gene and periodontitis, considering the severe and moderate severity levels, using dominant genetic model.

**ST2 gene**					
**Periodontitis**					
**Chromosome**	**SNV**	**Variant allele A1**	**OR**_**Adjusted**_ **(95%CI)**	**p**	**Permutation**
					**p**
2	rs148548829	C	0.63 (0.41–0.96)	0.03	0.03
2	rs10206753	G	0.63 (0.41–0.98)	0.04	0.04
**Moderate Periodontitis**					
**Chromosome**	**SNV**	**Variant allele A1**	**OR**_**Adjusted**_ **(95%CI)**	**p**	**Permutation**
					**p**
2	rs148548829	C	0.38 (0.16–0.92)	0.03	0.03
2	rs10206753	G	0.38 (0.16–0.92)	0.03	0.03
2	rs6751967	C	2.47 (1.22–5.01)	0.01	0.01
2	rs11693204	A	0.27 (0.08–0.92)	0.04	0.02
2	rs13017455	A	2.3 (1.15–4.76)	0.02	0.01
2	rs66780767	C	2.37 (1.16–4.82)	0.02	0.01
2	rs111533915	C	2.4 (1.19–4.85)	0.01	0.01
2	rs4988956	G	2.32 (1.14–4.71)	0.02	0.01
2	rs10192036	A	2.32 (1.14–4.72)	0.02	0.01
2	rs13019081	A	2.48 (1.23–5.03)	0.01	0.01
2	rs13011148	A	2.30 (1.13–4.66)	0.02	0.01
2	rs11123923	A	2.25 (1.11–4.56)	0.02	0.02
2	rs112593736	A	2.10 (1.05–4.23)	0.04	0.03
2	rs17026974	T	2.61 (1.01–6.71)	0.05	0.04
**Severe Periodontitis**					
**Chromosome**	**SNV**	**Variant allele A1**	**OR**_**Adjusted**_ **(95%CI)**	**p**	**Permutation**
					**p**
2	rs17639215	A	6.48 (1.00–41.63)	0.05	0.03
2	rs3771175	A	6.48 (1.00–41.63)	0.05	0.03
**IL33 gene**					
**Moderate Periodontitis**					
**Chromosome**	**SNV**	**Variant allele A1**	**OR**_**Adjusted**_ **(95%CI)**	**p**	**Permutation**
					**p**
9	rs2066362	A	0.47 (0.23–0.95)	0.03	0.04

OR_Adjusted_: odds ratio adjusted for total years of education, diagnosis of asthma, flossing, age, body mass index (BMI), mouth breathing habit, and PC1; 95%CI: confidence interval of 95%; p: statistical significance level (p≤0.05); Permutation p: statistical significance level of permutation (p≤0.05).

The relationship between the SNVs of the ST2 gene and periodontitis revealed an inverse association (OR <1) in two SNVs; the rs148548829 (C allele) and the rs10206753 (G allele).

This inverse association was again seen between these two SNVs of the ST2 gene and moderate periodontitis together with a third; rs11693204 (A allele).

On the other hand, a direct association between 11 SNVs (OR >1), of the ST2 gene: rs6751967 (C allele), rs13017455 (A allele), rs66780767 (C allele), rs111533915 (C allele), rs4988956 (G allele), rs10192036 (A allele), rs13019081 (A allele), rs13011148 (A allele), rs11123923 (A allele), rs112593736 (A allele), and rs17026974 (T allele), and moderate periodontitis was noted.

Similarly, a direct association (OR >1) between the rs17639215 (A allele) and the rs3771175 (A allele) of the ST2 gene and severe periodontitis was noted

Finally, an inverse association (OR <1) between the SNVs of the IL33 gene and moderate periodontitis was also observed with the SNV rs2066362 (A allele). All these models were adjusted for total years of education, diagnosis of asthma, flossing, age, BMI, mouth breathing habit and PC1 (Table 2).

Table 3 shows the adjusted associations between SNVs of the ST2 gene and *Aggregatibacter actinomycetemcomitans* relative amount, using the dominant genetic model. As can be seen, there was an inverse association with 9 SNVs (OR <1). In these models, only three covariables were used for adjustment: age, diagnosis of asthma, and PC1, since the number of observations in the models under analysis decreased.

**Table 3:** Adjusted association measurements, odds ratio and 95% confidence interval, between the SNVs of the ST2 gene and *Aggregatibacter actinomycetemcomitans* relative amount, considering dominant genetic model.

Chromosome	SNV	Variant allele A1	OR_Adjusted_ (95%CI)	p	Permutation
					p
2	rs111533915	C	0.42 (0.21–0.83)	0.01	0.01
2	rs6751967	**C**	0.43 (0.22–0.86)	0.02	0.02
2	rs13011148	A	0.44 (0.22–0.89)	0.02	0.02
2	rs13019081	A	0.49 (0.25–0.97)	0.04	0.03
2	rs112593736	A	0.5 (0.27–0.92)	0.03	0.02
2	rs66780767	C	0.46 (0.23–0.92)	0.03	0.02
2	rs11123923	A	0.49 (0.25–0.96)	0.04	0.03
2	rs4988956	G	0.50 (0.25–0.99)	0.05	0.03
2	rs10192036	A	0.50 (0.25–0.99)	0.05	0.03

OR_Adjusted_: odds ratio adjusted for age, diagnosis of asthma, and PC1; 95%CI: confidence interval of 95%; p: statistical significance level (p≤0.05); Permutation p: statistical significance level of permutation (p≤0.05).

**Fig 1:**
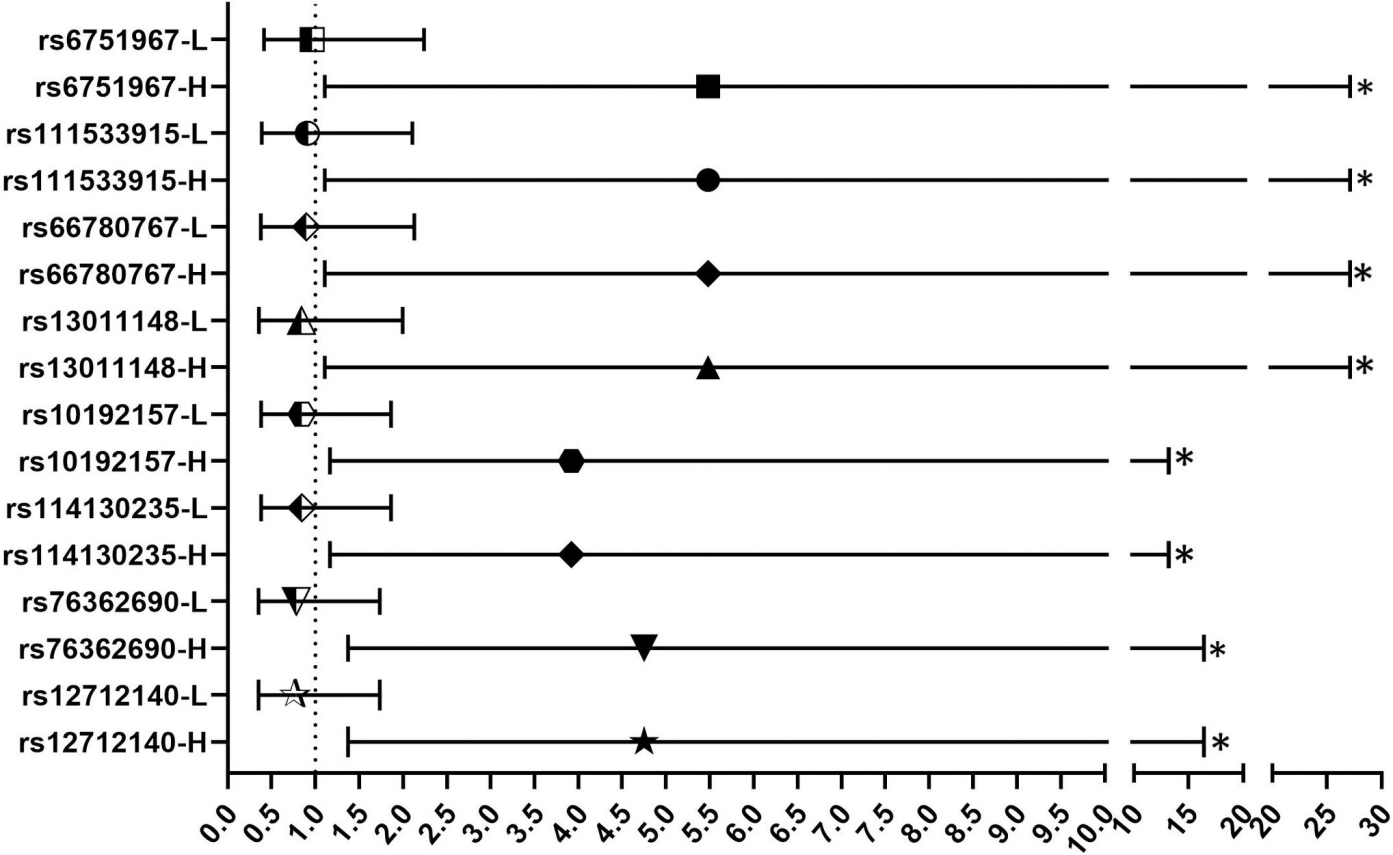
Adjusted association measurements, odds ratio (OR) and 95% confidence interval (95%CI), between the SNVs of the ST2 gene and periodontitis, through subgroup analysis, using the variable *Aggregatibacter actinomycetemcomitans* relative amount, showing the groups with high (H) and low (L) quantification of this bacteria in the biofilm and considering dominant genetic model. Adjusted for age, diagnosis of asthma, and PC1. *Statistical significance level: permutation p ≤ 0.05.

Fig 1 presents the results of the influence of the gene environment interaction on the association between the SNVs of the ST2 gene and periodontitis, through subgroup analysis using the variable *A*. *actinomycetemcomitans* relative amount. As can be noted, there was a direct association in 8 SNVs (OR >1) in the group with high numbers of this bacterium in the biofilm. In these models, only three covariables were used for adjustment: age, diagnosis of asthma, and PC1, for the same reason mentioned above. In the group with low numbers of *A*. *actinomycetemcomitans*, the measurements did not show any statistically significant associations.

**Fig 2:**
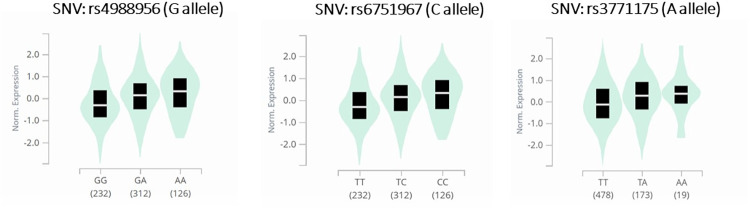
Expression quantitative trait locus (eQTL) analysis. SNVs that significantly decreased the expression in ST2 gene in human whole blood cells. A) The rs4988956 SNV (G allele), either in homozygosis, or in heterozygosis, induced lower gene expression when compared to A allele in homozygosis (p = 0.03). B) The rs6751967 SNV (C allele) led to an increase in the ST2 expression in homozygosis and in heterozygosis (p = 0.03). C) The rs3771175 SNV (A allele) in homozygosis led to an increase in the ST2 expression (p = 0.03).

Using the expression quantitative trait locus (eQTL) analysis (Fig 2), one SNV in ST2 gene was significantly decreased and two were significantly increased. The rs4988956 G allele, either in homozygosis, or in heterozygosis, resulted in lower gene expression compared with the A allele in homozygosis (p = 0.03) (Fig 2A). The rs6751967 C allele and the rs3771175 A allele, either in homozygosis, or in heterozygosis, led to an increase in the ST2 expression (Fig 2B and 2C), (p = 0.03).

**Fig 3:**
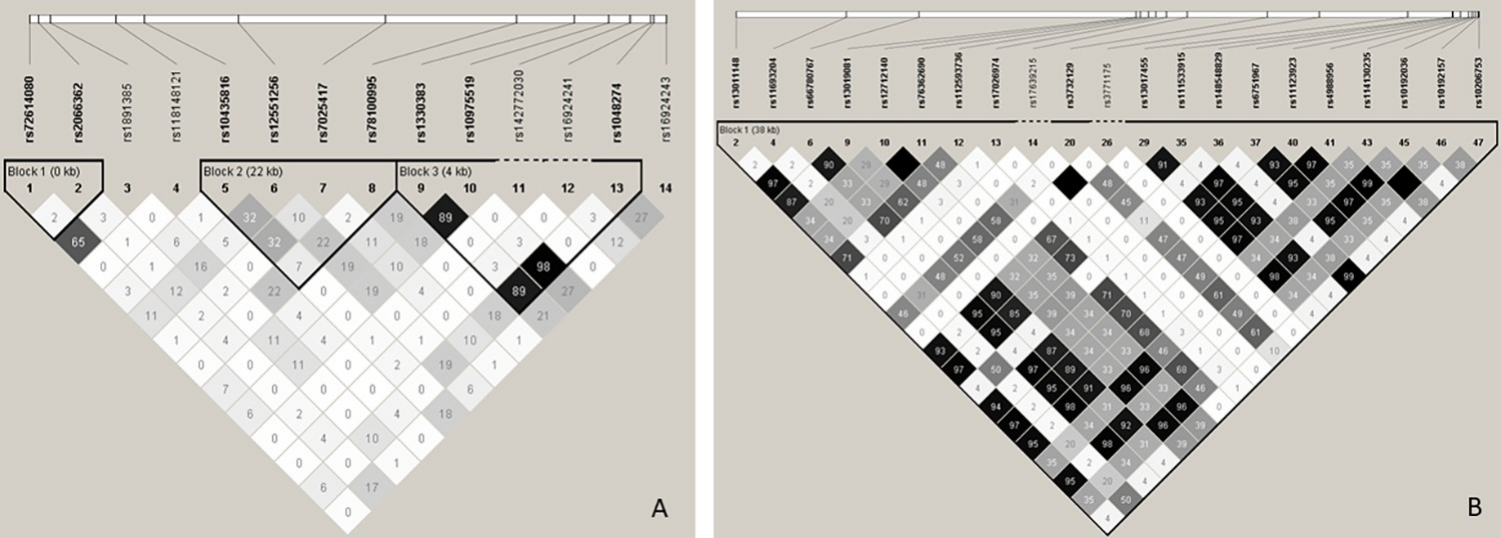
Linkage disequilibrium analysis presenting studied SNVs of IL-33 (A) and ST2 (B) in high or complete linkage disequilibrium.

The linkage disequilibrium analysis (Fig 3) showed that many of the SNVs studied were in high or complete linkage disequilibrium. Some of them are worth mentioning, in combination with the regulome characterization. For example, in ST2 gene, the rs148548829 SNV was classified as 3a score, and it is in complete linkage disequilibrium with the missense rs10206753 variant (r^2^ = 0.99). The rs4988956 SNV was classified as 3a score and is in linkage disequilibrium with the rs6751967 SNV (r^2^ = 0.95), with rs10192036 SNV (r^2^ = 0.99) and with rs11123923 SNV (r^2^ = 0.97). The rs13017455 SNV is in high linkage disequilibrium with rs7996072 SNV; the rs3771175 SNV is in complete linkage disequilibrium (r^2^ = 1) with the rs17639215 SNV which was classified as 2a score.

## Discussion

Periodontitis is a highly prevalent worldwide chronic disease. The severity of which varies according to largely still unknown genetic factors. In this context, individuals diagnosed with periodontitis may present with different disease expressions and progression as a result of differences in each individual host genetics. To contribute to the understanding of this topic, this study focused on the investigation of SNVs in the IL33 gene and the ST2 gene since they have been implicated in the host’s immune response in periodontitis [37, 38].

The main findings showed both inverse and direct associations between SNVs of both genes with moderate and severe periodontitis. There is absence of previous genetic studies regarding the influence of IL-33 and ST2 gene variants that allows comparisons with the present findings.

Additionally, in a subgroup analysis, our results showed an association between 9 SNVs of the ST2 gene and the relative amount of *Aggregatibacter actinomycetemcomitans*. Finally, the present study has highlighted the influence of the gene environment interaction, between 8 SNVs of the ST2 gene and periodontitis in the subgroup with high *A*. *actinomycetemcomitans* relative amount. This was not observed in the low *Aa* subgroup. The significance of these findings in terms of the individual host response in the pathogenesis of periodontitis is based on the understanding that each SNV can determine different functional outcomes. No studies have yet investigated the relationship between this pathogen and genetic variants in the IL-33/ST2 axis.

In the present study, complementary findings from the in silico analysis showed that the SNVs of the ST2 gene, that were associated with periodontitis (one with moderate periodontitis and two with severe periodontitis) had a functional impact that was reflected in the expression of the ST2 gene in human whole blood cells. Thus, reinforcing the hypothesis that these SNVs could have a relevant role in the pathogenesis of periodontitis, through the alteration of the IL-33/ST2 axis. In other words, the alteration in circulating ST2 levels in an individual diagnosed with periodontitis could lead to an increase in available IL-33 and, consequently, it could favor bone resorption and contribute to an increase in the severity of periodontitis [38, 39].

Additionally, the analysis of linkage disequilibrium and regulome indicated that other SNVs in the ST2 gene associated with periodontitis are missense, that is, a single variant is capable to codify an amino acid different from the usual amino acid present in most of the population, which may alter the protein structure and, consequently, its function.

Furthermore, some SNVs in the ST2 gene associated with periodontitis were in linkage disequilibrium with other missense or other intronic SNVs. This correlation between nearby SNVs allows them to assume similar characteristics among themselves. For example, in linkage disequilibrium with other missense SNVs, the function of the ST2 gene could be altered. On the other hand, linkage disequilibrium with other intronic SNV, could lead to an impairment in the regulation of gene expression, altering RNA transcription, hence compromising ST2 gene production with a potential impact on the pathogenesis of periodontitis [40].

There are however some limitations to the present study. The use of the dominant model for the genetic analysis, which was due to the better results found for the investigated SNVs, allowed for differences in the functional analysis to be observed. Furthermore, the cross-sectional study design employed did not allow us to establish a causal relationship between the investigated SNVs and the natural history of periodontitis. In addition, due to the novelty of this line of research, it is possible that other factors, not measured, may have exerted some degree of influence over the investigated associations and were not used as confounders in the analysis of the adjusted association measurements.

Another limitation was the reduced size of individuals in the subgroups, based on the relative quantification of Aa, as this stratification may have influenced the power of the study, causing a decrease in precision or loss of statistical significance of the association measurement in some analyses. Additionally, the production of proteins in samples from study participants was not investigated to confirm the impact of SNVs on phenotypic characteristics. This however was mitigated by the in silico analyzes which enabled us to clarify the functional impact of the referred SNVs.

An issue to be highlighted is the fact that some SNVs were associated with only one level of periodontitis severity, and, on the other hand, this association was not statistically significant for the model between the SNV and periodontitis. This was since different analysis models were performed in the study considering only those participants with a specific diagnosis of periodontitis severity level. In the model between the SNV and periodontitis, all participants with mild, moderate, and severe levels of periodontitis were included, interfering with the final finding of the association measurement.

Among the strengths of this study was the quantification of *A*. *actinomycetemcomitans* in the subgingival biofilm. The presence of this pathogen has been strongly related to periodontitis pathogenesis, being indicative of a dysbiotic subgingival biofilm. These findings reinforce the gene-environment interaction, that is, between host genetic factors and oral microbial colonization.

The establishment of a genetic profile applicable to periodontitis should prove valuable to the understanding of the pathogenesis of this disease, particularly with respect to the role(s) played by host susceptibility factors.

## Conclusion

An association was observed between an IL-33 SNV and 14 ST2 SNVs and periodontitis, suggesting that the different tissue responses among individuals with periodontitis may be influenced by the genetics of the host, altering the expression of genes. These exploratory findings indicate the need for future investigations with the creation of new hypotheses and thus, better clarifying the role of these proteins in the host response in the pathogenesis of periodontitis, which may contribute to the individuality of disease expression and progression.

## Supporting information

S1 Table Characterization of the analyzed single nucleotide variants (SNVs), including the ones that were associated with periodontitis in the present study (in bold). (DOCX)

S2 Table Adjusted association measurements, odds ratio and 95% confidence interval, between the SNVs of the IL-33 gene and periodontitis, as well as the SNVs of the ST2 gene and periodontitis, considering the severe and moderate severity levels, using additive (ADD), dominant (DOM) and recessive (REC) genetic model. (DOCX)

S3 Table Adjusted association measurements, odds ratio and 95% confidence interval, between the SNVs of the ST2 gene and IL33 gene, and *Aggregatibacter actinomycetemcomitans* relative amount, considering additive (ADD), dominant (DOM) and recessive (REC) genetic model. (DOCX)
