# Supplementary material for: Single nucleotide variants in the IL33 and IL1RL1 (ST2) genes are associated with periodontitis and with Aggregatibacter actinomycetemcomitans in the dental plaque biofilm: A putative role in understanding the host immune response in periodontitis
Source: PLoS One. 2023 Mar 22;18(3):e0283179. doi: 10.1371/journal.pone.0283179 (PMC10032506; doi:10.1371/journal.pone.0283179)
Supplement: S1 Table — (DOCX) [file pone.0283179.s001.docx]

**S1 Table.** Characterization of the analyzed single nucleotide variants (SNVs), including the ones that were associated with periodontitis in the present study (in bold)

| **Chromosome** | **SNV** | **Minor allele** | **Ancestral allele** | **Minor Allele Frequency** | **Location / Function** | **Regulome DB** |
| --- | --- | --- | --- | --- | --- | --- |
| **IL33 gene** | | | | | | |
| 9 | rs72614080 | A | G | 0.06 | Intron | 2b |
| 9 | rs1891385 | C | A | 0.08 | Intron | 3a |
| **9** | **rs2066362** | **A** | **C** | **0.31** | **Intron** | **4** |
| 9 | rs7025417 | G | A | 0.19 | Intron | 4 |
| 9 | rs1048274 | A | G | 0.37 | 3 Prime Untranslated Region Variant | 5 |
| 9 | rs118148121 | T | A | 0.02 | Intron | 5 |
| 9 | rs142772030 | A | G | 0.01 | Intron | 5 |
| 9 | rs78100995 | C | G | 0.1 | Intron | 5 |
| 9 | rs10975519 | A | G | 0.37 | Synonymous Variant | 6 |
| 9 | rs16924243 | G | A | 0.14 | 3 Prime Untranslated Region Variant | 7 |
| 9 | rs10435816 | G | A | 0.42 | Intron | 7 |
| 9 | rs12551256 | G | A | 0.33 | Intron | 7 |
| 9 | rs1330383 | A | C | 0.36 | Intron | 7 |
| 9 | rs16924241 | G | C | 0.02 | Missense | 7 |
| **ST2 gene** | | | | | | |
| 2 | rs3771180 | C | A | 0.46 | Intron | 2b |
| **2** | **rs17639215** | **A** | **G** | **0.01** | **Intron** | **2b** |
| 2 | rs73944273 | A | G | 0.01 | Intron | 2b |
| **2** | **rs4988956** | **G** | **A** | **0.46** | **Missense** | **3a** |
| 2 | rs1420101 | G | A | 0.46 | Non Coding Transcript Variant | 3a |
| 2 | rs12712140 | A | T | 0.36 | Intron | 3a |
| 2 | rs114130235 | A | G | 0.35 | Missense | 3a |
| 2 | rs13408661 | A | C | 0.27 | Intron | 3a |
| 2 | rs35298562 | C | A | 0.27 | Synonymous | 3a |
| **2** | **rs148548829** | **C** | **A** | **0.18** | **Intron** | **3a** |
| 2 | rs1420101 | G | A | 0.46 | Non Coding Transcript Variant | 3a |
| 2 | rs13408661 | A | C | 0.27 | Intron | 3a |
| 2 | rs35298562 | C | A | 0.27 | Synonymous Variant | 3a |
| 2 | rs12999517 | G | A | 0.46 | Intron | 4 |
| 2 | rs873022 | A | T | 0.46 | Intron | 4 |
| 2 | rs950880 | A | G | 0.46 | Intron | 4 |
| 2 | rs12905 | A | C | 0.46 | Non Coding Transcript Variant | 4 |
| 2 | rs10192157 | A | G | 0.35 | Missense | 4 |
| 2 | rs3732129 | A | G | 0.26 | Intron | 4 |
| **2** | **rs112593736** | **A** | **G** | **0.21** | **Intron** | **4** |
| **2** | **rs10206753** | **G** | **A** | **0.18** | **Missense** | **4** |
| **2** | **rs111533915** | **C** | **G** | **0.16** | **Intron** | **4** |
| **2** | **rs13011148** | **A** | **G** | **0.16** | **Intron** | **4** |
| **2** | **rs10192036** | **A** | **C** | **0.16** | **Missense** | **4** |
| **2** | **rs17026974** | **T** | **A** | **0.06** | **Intron** | **4** |
| 2 | rs3821204 | G | A | 0.06 | Non Coding Transcript Variant | 4 |
| 2 | rs6543119 | C | G | 0.05 | Intron | 4 |
| **2** | **rs3771175** | **A** | **G** | **0.01** | **Non Coding Transcript Variant** | **4** |
| 2 | rs10197862 | A | G | 0.46 | Intron | 5 |
| 2 | rs1420103 | G | A | 0.46 | Intron | 5 |
| 2 | rs76362690 | A | G | 0.36 | Intron | 5 |
| 2 | rs12469506 | A | G | 0.2 | Intron | 5 |
| 2 | rs17027006 | A | C | 0.2 | Intron | 5 |
| 2 | rs6704565 | G | A | 0.2 | Intron | 5 |
| **2** | **rs11123923** | **A** | **C** | **0.16** | **Intron** | **5** |
| **2** | **rs13019081** | **A** | **G** | **0.16** | **Intron** | **5** |
| **2** | **rs66780767** | **C** | **G** | **0.16** | **Intron** | **5** |
| **2** | **rs6751967** | **A** | **G** | **0.16** | **Intron** | **5** |
| 2 | rs3771177 | A | C | 0.15 | Intron | 5 |
| 2 | rs12999542 | C | G | 0.03 | Intron | 5 |
| 2 | rs76887186 | G | A | 0.03 | Intron | 5 |
| 2 | rs114797672 | A | G | 0.02 | Missense | 5 |
| 2 | rs72823641 | T | A | 0.20 | Intron | 6 |
| **2** | **rs11693204** | **A** | **G** | **0.10** | **Intron** | **6** |
| 2 | rs12479210 | A | C | 0.26 | Intron | 7 |
| **2** | **rs13017455** | **A** | **G** | **0.16** | **Intron** | **7** |
| 2 | rs55927292 | A | G | 0.07 | Intron | 7 |
