# Supplementary material for: Single nucleotide variants in the IL33 and IL1RL1 (ST2) genes are associated with periodontitis and with Aggregatibacter actinomycetemcomitans in the dental plaque biofilm: A putative role in understanding the host immune response in periodontitis
Source: PLoS One. 2023 Mar 22;18(3):e0283179. doi: 10.1371/journal.pone.0283179 (PMC10032506; doi:10.1371/journal.pone.0283179)
Supplement: S2 Table — (DOCX) [file pone.0283179.s002.docx]

**S2 Table.** Adjusted association measurements, odds ratio and 95% confidence interval, between the SNVs of the IL-33 gene and periodontitis, as well as the SNVs of the ST2 gene and periodontitis, considering the severe and moderate severity levels, using additive (ADD), dominant (DOM) and recessive (REC) genetic model

| ***ST2* gene** | | | | | | | | | | |
| --- | --- | --- | --- | --- | --- | --- | --- | --- | --- | --- |
| \| **Periodontitis** \| \| --- \| | | | | | | | | | | |
| **CHR** | **SNV** | **Variant allele A1** | **Model** | | **OR_Adjusted_ (95%CI)** | | | | **p-value** | |
| 2 | rs17639215 | A | ADD | | 5.752 | (1.092 | | 30.3) | 0.03907 | |
| 2 | rs17639215 | A | ADD | | 5.752 | (1.092 | | 30.3) | 0.03907 | |
| 2 | rs3771175 | A | ADD | | 5.752 | (1.092 | | 30.3) | 0.03907 | |
| 2 | rs17026974 | T | ADD | | 1.636 | (0.8725 | | 3.068) | 0.1248 | |
| 2 | rs950880 | A | ADD | | 0.7915 | (0.5704 | | 1.098) | 0.1618 | |
| 2 | rs1420103 | G | ADD | | 0.7915 | (0.5704 | | 1.098) | 0.1618 | |
| 2 | rs3771180 | C | ADD | | 0.7915 | (0.5704 | | 1.098) | 0.1618 | |
| 2 | rs1420101 | G | ADD | | 0.7915 | (0.5704 | | 1.098) | 0.1618 | |
| 2 | rs12999517 | G | ADD | | 0.7915 | (0.5704 | | 1.098) | 0.1618 | |
| 2 | rs10197862 | A | ADD | | 0.7915 | (0.5704 | | 1.098) | 0.1618 | |
| 2 | rs4988956 | G | ADD | | 0.7915 | (0.5704 | | 1.098) | 0.1618 | |
| 2 | rs76887186 | G | ADD | | 1.842 | (0.7554 | | 4.489) | 0.1793 | |
| 2 | rs1420101 | A | ADD | | 1.786 | (0.7642 | | 4.172) | 0.1806 | |
| 2 | rs114797672 | A | ADD | | 0.4985 | (0.1745 | | 1.424) | 0.1936 | |
| 2 | rs3771177 | A | ADD | | 0.7476 | (0.4688 | | 1.192) | 0.2219 | |
| 2 | rs17027006 | A | ADD | | 1.258 | (0.8457 | | 1.871) | 0.2572 | |
| 2 | rs72823641 | T | ADD | | 1.246 | (0.8379 | | 1.853) | 0.2772 | |
| 2 | rs12469506 | A | ADD | | 1.246 | (0.8379 | | 1.853) | 0.2772 | |
| 2 | rs6704565 | G | ADD | | 1.246 | (0.8379 | | 1.853) | 0.2772 | |
| 2 | rs10206753 | G | ADD | | 0.8136 | (0.5374 | | 1.232) | 0.3297 | |
| 2 | rs114130235 | G | ADD | | 1.504 | (0.6416 | | 3.527) | 0.3477 | |
| 2 | rs3821204 | G | ADD | | 0.6785 | (0.2924 | | 1.575) | 0.3666 | |
| 2 | rs148548829 | C | ADD | | 0.8319 | (0.5509 | | 1.256) | 0.3816 | |
| 2 | rs11693204 | A | ADD | | 0.8103 | (0.4815 | | 1.363) | 0.4281 | |
| 2 | rs73944273 | A | ADD | | 0.6608 | (0.2207 | | 1.978) | 0.459 | |
| 2 | rs12905 | A | ADD | | 1.123 | (0.8146 | | 1.548) | 0.4789 | |
| 2 | rs13408661 | A | ADD | | 0.8824 | (0.6102 | | 1.276) | 0.5064 | |
| 2 | rs13019081 | A | ADD | | 1.146 | (0.7504 | | 1.749) | 0.5288 | |
| 2 | rs873022 | A | ADD | | 1.099 | (0.797 | | 1.516) | 0.5641 | |
| 2 | rs12712140 | A | ADD | | 0.9396 | (0.6712 | | 1.315) | 0.7163 | |
| 2 | rs76362690 | A | ADD | | 0.9396 | (0.6712 | | 1.315) | 0.7163 | |
| 2 | rs66780767 | C | ADD | | 1.081 | (0.7037 | | 1.66)) | 0.7222 | |
| 2 | rs114130235 | A | ADD | | 0.9456 | (0.6739 | | 1.327 | 0.7463 | |
| 2 | rs10192157 | A | ADD | | 0.9456 | (0.6739 | | 1.327) | 0.7463 | |
| 2 | rs6751967 | A | ADD | | 1.068 | (0.6967 | | 1.638) | 0.7622 | |
| 2 | rs55927292 | A | ADD | | 0.9032 | (0.4459 | | 1.829) | 0.7774 | |
| 2 | rs4988956 | G | ADD | | 1.056 | (0.6885 | | 1.62) | 0.8024 | |
| 2 | rs10192036 | A | ADD | | 1.056 | (0.6885 | | 1.62)) | 0.8024 | |
| 2 | rs13017455 | A | ADD | | 1.045 | (0.6783 | | 1.61) | 0.8414 | |
| 2 | rs13011148 | A | ADD | | 1.033 | (0.6761 | | 1.578) | 0.8806 | |
| 2 | rs12479210 | A | ADD | | 0.9772 | (0.6772 | | 1.41) | 0.902 | |
| 2 | rs12999542 | C | ADD | | 0.9391 | (0.3368 | | 2.618) | 0.9044 | |
| 2 | rs112593736 | A | ADD | | 1.02 | (0.6977 | | 1.492) | 0.9175 | |
| 2 | rs111533915 | C | ADD | | 1.022 | (0.6699 | | 1.558) | 0.9208 | |
| 2 | rs11123923 | A | ADD | | 1.01 | (0.662 | | 1.542) | 0.9617 | |
| 2 | rs3732129 | A | ADD | | 0.9922 | (0.6878 | | 1.431) | 0.9668 | |
| 2 | rs6543119 | C | ADD | | 1.01 | (0.4822 | | 2.117) | 0.9781 | |
| 2 | rs35298562 | C | ADD | | 0.9995 | (0.6939 | | 1.44) | 0.9978 | |
| 2 | rs11693204 | A | DOM | | 0.6534 | (0.395 | | 1.081) | 0.09741 | |
| 2 | rs3771177 | A | DOM | | 0.7327 | (0.4667 | | 1.15) | 0.1764 | |
| 2 | rs17026974 | T | DOM | | 1.53 | (0.7963 | | 2.94) | 0.2019 | |
| 2 | rs13011148 | A | DOM | | 1.329 | (0.8383 | | 2.107) | 0.2264 | |
| 2 | rs11123923 | A | DOM | | 1.307 | (0.8266 | | 2.067) | 0.2521 | |
| 2 | rs13019081 | A | DOM | | 1.286 | (0.8142 | | 2.031) | 0.2808 | |
| 2 | rs17027006 | A | DOM | | 1.258 | (0.8261 | | 1.916) | 0.2846 | |
| 2 | rs72823641 | T | DOM | | 1.255 | (0.8238 | | 1.912) | 0.2903 | |
| 2 | rs12469506 | A | DOM | | 1.255 | (0.8238 | | 1.912) | 0.2903 | |
| 2 | rs6704565 | G | DOM | | 1.255 | (0.8238 | | 1.912) | 0.2903 | |
| 2 | rs73944273 | A | DOM | | 0.5798 | (0.2096 | | 1.604) | 0.2939 | |
| 2 | rs111533915 | C | DOM | | 1.273 | (0.807 | | 2.007) | 0.2994 | |
| 2 | rs66780767 | C | DOM | | 1.278 | (0.8038 | | 2.033) | 0.2996 | |
| 2 | rs13017455 | A | DOM | | 1.278 | (0.804 | | 2.031) | 0.2996 | |
| 2 | rs112593736 | A | DOM | | 1.246 | (0.815 | | 1.906) | 0.3095 | |
| 2 | rs4988956 | G | DOM | | 1.257 | (0.7927 | | 1.994) | 0.3307 | |
| 2 | rs10192036 | A | DOM | | 1.257 | (0.7927 | | 1.994) | 0.3307 | |
| 2 | rs17639215 | A | DOM | | 2.321 | (0.405 | | 13.3) | 0.3445 | |
| 2 | rs3771175 | A | DOM | | 2.321 | (0.405 | | 13.3) | 0.3445 | |
| 2 | rs12712140 | A | DOM | | 0.8281 | (0.5464 | | 1.255) | 0.3741 | |
| 2 | rs76362690 | A | DOM | | 0.8281 | (0.5464 | | 1.255) | 0.3741 | |
| 2 | rs6751967 | A | DOM | | 1.224 | (0.7739 | | 1.937) | 0.3873 | |
| 2 | rs12999542 | C | DOM | | 0.6847 | (0.273 | | 1.717) | 0.4195 | |
| 2 | rs950880 | A | DOM | | 0.8326 | (0.529 | | 1.311) | 0.4287 | |
| 2 | rs1420103 | G | DOM | | 0.8326 | (0.529 | | 1.311) | 0.4287 | |
| 2 | rs3771180 | C | DOM | | 0.8326 | (0.529 | | 1.311) | 0.4287 | |
| 2 | rs1420101 | G | DOM | | 0.8326 | (0.529 | | 1.311) | 0.4287 | |
| 2 | rs12999517 | G | DOM | | 0.8326 | (0.529 | | 1.311) | 0.4287 | |
| 2 | rs10197862 | A | DOM | | 0.8326 | (0.529 | | 1.311) | 0.4287 | |
| 2 | rs4988956 | G | DOM | | 0.8326 | (0.529 | | 1.311) | 0.4287 | |
| 2 | rs13408661 | A | DOM | | 0.8501 | (0.5649 | | 1.279) | 0.436 | |
| 2 | rs873022 | A | DOM | | 1.16 | (0.7408 | | 1.817) | 0.5163 | |
| 2 | rs12905 | A | DOM | | 1.16 | (0.7408 | | 1.817) | 0.5163 | |
| 2 | rs6543119 | C | DOM | | 1.23 | (0.6132 | | 2.466) | 0.5603 | |
| 2 | rs114130235 | A | DOM | | 0.9022 | (0.5967 | | 1.364) | 0.6257 | |
| 2 | rs10192157 | A | DOM | | 0.9022 | (0.5967 | | 1.364) | 0.6257 | |
| 2 | rs3732129 | A | DOM | | 0.9114 | (0.6056 | | 1.372) | 0.6565 | |
| 2 | rs55927292 | A | DOM | | 1.148 | (0.6118 | | 2.154) | 0.6676 | |
| 2 | rs35298562 | C | DOM | | 0.917 | (0.609 | | 1.381) | 0.678 | |
| 2 | rs12479210 | A | DOM | | 0.9546 | (0.6344 | | 1.436) | 0.8235 | |
| 2 | rs3821204 | G | DOM | | 1.072 | (0.5467 | | 2.103) | 0.8393 | |
| 2 | rs114130235 | G | DOM | | 1.088 | (0.4674 | | 2.532) | 0.8449 | |
| 2 | rs114797672 | A | DOM | | 1.056 | (0.4408 | | 2.528) | 0.9031 | |
| 2 | rs76887186 | G | DOM | | 1.041 | (0.4064 | | 2.667) | 0.9333 | |
| 2 | rs1420101 | A | DOM | | 0.9646 | (0.3954 | | 2.353) | 0.9369 | |
| 2 | rs13019081 | A | REC | | 0.1952 | (0.01987 | | 1.918) | 0.1611 | |
| 2 | rs12479210 | A | REC | | 0.549 | (0.1924 | | 1.566) | 0.2623 | |
| 2 | rs3732129 | A | REC | | 0.549 | (0.1924 | | 1.566) | 0.2623 | |
| 2 | rs17027006 | A | REC | | 1.856 | (0.6096 | | 5.652) | 0.2763 | |
| 2 | rs72823641 | T | REC | | 1.855 | (0.6091 | | 5.651) | 0.2768 | |
| 2 | rs12469506 | A | REC | | 1.855 | (0.6091 | | 5.651) | 0.2768 | |
| 2 | rs6704565 | G | REC | | 1.855 | (0.6091 | | 5.651) | 0.2768 | |
| 2 | rs11123923 | A | REC | | 0.4242 | (0.08377 | | 2.148) | 0.3001 | |
| 2 | rs13011148 | A | REC | | 0.4321 | (0.08493 | | 2.198) | 0.312 | |
| 2 | rs111533915 | C | REC | | 0.4321 | (0.08493 | | 2.198) | 0.312 | |
| 2 | rs950880 | A | REC | | 0.7536 | (0.4265 | | 1.332) | 0.33 | |
| 2 | rs1420103 | G | REC | | 0.7536 | (0.4265 | | 1.332) | 0.33 | |
| 2 | rs3771180 | C | REC | | 0.7536 | (0.4265 | | 1.332) | 0.33 | |
| 2 | rs1420101 | G | REC | | 0.7536 | (0.4265 | | 1.332) | 0.33 | |
| 2 | rs12999517 | G | REC | | 0.7536 | (0.4265 | | 1.332) | 0.33 | |
| 2 | rs10197862 | A | REC | | 0.7536 | (0.4265 | | 1.332) | 0.33 | |
| 2 | rs4988956 | G | REC | | 0.7536 | (0.4265 | | 1.332) | 0.33 | |
| 2 | rs13408661 | A | REC | | 0.6446 | (0.2407 | | 1.726) | 0.3824 | |
| 2 | rs4988956 | G | REC | | 0.4855 | (0.09398 | | 2.508) | 0.3883 | |
| 2 | rs10192036 | A | REC | | 0.4855 | (0.09398 | | 2.508) | 0.3883 | |
| 2 | rs66780767 | C | REC | | 0.4961 | (0.09548 | | 2.578) | 0.4044 | |
| 2 | rs6751967 | A | REC | | 0.4961 | (0.09548 | | 2.578) | 0.4044 | |
| 2 | rs13017455 | A | REC | | 0.505 | (0.09671 | | 2.637) | 0.4178 | |
| 2 | rs17026974 | T | REC | | 2.629 | (0.2175 | | 31.77) | 0.4471 | |
| 2 | rs35298562 | C | REC | | 0.702 | (0.2635 | | 1.87) | 0.4792 | |
| 2 | rs114130235 | A | REC | | 0.7801 | (0.3789 | | 1.606) | 0.5004 | |
| 2 | rs10192157 | A | REC | | 0.7801 | (0.3789 | | 1.606) | 0.5004 | |
| 2 | rs3771177 | A | REC | | 0.5985 | (0.1118 | | 3.205) | 0.5487 | |
| 2 | rs12712140 | A | REC | | 0.8082 | (0.4006 | | 1.631) | 0.5522 | |
| 2 | rs76362690 | A | REC | | 0.8082 | (0.4006 | | 1.631) | 0.5522 | |
| 2 | rs11693204 | A | REC | | 1.506 | (0.2278 | | 9.955) | 0.671 | |
| 2 | rs112593736 | A | REC | | 0.7938 | (0.2691 | | 2.342) | 0.6757 | |
| 2 | rs873022 | A | REC | | 0.9066 | (0.5249 | | 1.566) | 0.7252 | |
| 2 | rs148548829 | C | REC | | 0.8339 | (0.2473 | | 2.812) | 0.7697 | |
| 2 | rs10206753 | G | REC | | 0.8339 | (0.2473 | | 2.812) | 0.7697 | |
| 2 | rs12905 | A | REC | | 0.9654 | (0.562 | | 1.658) | 0.8984 | |
| **Moderate Periodontitis** | | | | | | | | | | |
| 2 | rs148548829 | C | ADD | | 0.3876 | (0.1676 | | 0.8963) | 0.0267 | |
| 2 | rs10206753 | G | ADD | | 0.3884 | (0.1679 | | 0.8985) | 0.0271 | |
| 2 | rs6751967 | A | ADD | | 1.891 | (1.057 | | 3.385) | 0.03183 | |
| 2 | rs11693204 | A | ADD | | 0.2713 | (0.07948 | | 0.9259) | 0.03727 | |
| 2 | rs13017455 | A | ADD | | 1.839 | (1.02 | | 3.316) | 0.04292 | |
| 2 | rs66780767 | C | ADD | | 1.829 | (1.018 | | 3.284) | 0.0434 | |
| 2 | rs111533915 | C | ADD | | 1.799 | (1.014 | | 3.19) | 0.04452 | |
| 2 | rs4988956 | G | ADD | | 1.795 | (1 | | 3.219) | 0.04987 | |
| 2 | rs10192036 | A | ADD | | 1.795 | (1 | | 3.219) | 0.04987 | |
| 2 | rs112593736 | A | ADD | | 1.691 | (0.9931 | | 2.881) | 0.05305 | |
| 2 | rs13011148 | A | ADD | | 1.74 | (0.9775 | | 3.097) | 0.05976 | |
| 2 | rs13019081 | A | ADD | | 1.748 | (0.9692 | | 3.151) | 0.06347 | |
| 2 | rs11123923 | A | ADD | | 1.71 | (0.9615 | | 3.042) | 0.06779 | |
| 2 | rs17026974 | T | ADD | | 2.213 | (0.9287 | | 5.275) | 0.07297 | |
| 2 | rs17639215 | A | ADD | | 8.46 | (0.7634 | | 93.75) | 0.08186 | |
| 2 | rs3771175 | A | ADD | | 8.46 | (0.7634 | | 93.75) | 0.08186 | |
| 2 | rs1420101 | A | ADD | | 2.645 | 0.8752 | | 7.992) | 0.08476 | |
| 2 | rs76887186 | G | ADD | | 2.434 | (0.7281 | | 8.136) | 0.1486 | |
| 2 | rs114797672 | A | ADD | | 0.274 | (0.03442 | | 2.181) | 0.2212 | |
| 2 | rs3771177 | A | ADD | | 0.6213 | (0.2805 | | 1.376) | 0.2408 | |
| 2 | rs72823641 | T | ADD | | 1.29 | (0.6943 | | 2.396) | 0.4208 | |
| 2 | rs17027006 | A | ADD | | 1.29 | (0.6943 | | 2.396) | 0.4208 | |
| 2 | rs12469506 | A | ADD | | 1.29 | (0.6943 | | 2.396) | 0.4208 | |
| 2 | rs6704565 | G | ADD | | 1.29 | (0.6943 | | 2.396) | 0.4208 | |
| 2 | rs12905 | A | ADD | | 1.202 | (0.7338 | | 1.968) | 0.4655 | |
| 2 | rs873022 | A | ADD | | 1.134 | (0.6924 | | 1.858) | 0.6169 | |
| 2 | rs114130235 | A | ADD | | 0.8964 | (0.5326 | | 1.509) | 0.6805 | |
| 2 | rs10192157 | A | ADD | | 0.8964 | (0.5326 | | 1.509) | 0.6805 | |
| 2 | rs114130235 | G | ADD | | 1.241 | (0.322 | | 4.786) | 0.7534 | |
| 2 | rs35298562 | C | ADD | | 1.09 | (0.6281 | | 1.893) | 0.7586 | |
| 2 | rs12712140 | A | ADD | | 0.9261 | (0.5549 | | 1.545) | 0.7688 | |
| 2 | rs76362690 | A | ADD | | 0.9261 | (0.5549 | | 1.545) | 0.7688 | |
| 2 | rs13408661 | A | ADD | | 1.077 | (0.6236 | | 1.861) | 0.7896 | |
| 2 | rs6543119 | C | ADD | | 1.141 | (0.381 | | 3.417) | 0.8136 | |
| 2 | rs55927292 | A | ADD | | 1.115 | (0.4092 | | 3.038) | 0.8315 | |
| 2 | rs73944273 | A | ADD | | 0.858 | (0.1754 | | 4.197) | 0.8501 | |
| 2 | rs950880 | A | ADD | | 0.9692 | (0.5868 | | 1.601) | 0.9029 | |
| 2 | rs1420103 | G | ADD | | 0.9692 | (0.5868 | | 1.601) | 0.9029 | |
| 2 | rs3771180 | C | ADD | | 0.9692 | (0.5868 | | 1.601) | 0.9029 | |
| 2 | rs1420101 | G | ADD | | 0.9692 | (0.5868 | | 1.601) | 0.9029 | |
| 2 | rs12999517 | G | ADD | | 0.9692 | (0.5868 | | 1.601) | 0.9029 | |
| 2 | rs10197862 | A | ADD | | 0.9692 | (0.5868 | | 1.601) | 0.9029 | |
| 2 | rs4988956 | G | ADD | | 0.9692 | (0.5868 | | 1.601) | 0.9029 | |
| 2 | rs3821204 | G | ADD | | 1.065 | (0.343 | | 3.308) | 0.9131 | |
| 2 | rs12479210 | A | ADD | | 1.016 | (0.5809 | | 1.776) | 0.9565 | |
| 2 | rs3732129 | A | ADD | | 1.014 | (0.5797 | | 1.774) | 0.9605 | |
| 2 | rs873022 | A | DOM | | 2.305 | (0.9116 | | 5.829) | 0.07766 | |
| 2 | rs12905 | A | DOM | | 2.305 | (0.9116 | | 5.829) | 0.07766 | |
| 2 | rs17639215 | A | DOM | | 8.46 | (0.7634 | | 93.75) | 0.08186 | |
| 2 | rs3771175 | A | DOM | | 8.46 | (0.7634 | | 93.75) | 0.08186 | |
| 2 | rs1420101 | A | DOM | | 2.645 | (0.8752 | | 7.992) | 0.08476 | |
| 2 | rs76887186 | G | DOM | | 2.434 | (0.7281 | | 8.136) | 0.1486 | |
| 2 | rs114797672 | A | DOM | | 0.274 | (0.03442 | | 2.181) | 0.2212 | |
| 2 | rs3771177 | A | DOM | | 0.6492 | (0.2786 | | 1.513) | 0.3169 | |
| 2 | rs72823641 | T | DOM | | 1.36 | (0.6742 | | 2.742) | 0.3907 | |
| 2 | rs17027006 | A | DOM | | 1.36 | (0.6742 | | 2.742) | 0.3907 | |
| 2 | rs12469506 | A | DOM | | 1.36 | (0.6742 | | 2.742) | 0.3907 | |
| 2 | rs6704565 | G | DOM | | 1.36 | (0.6742 | | 2.742) | 0.3907 | |
| 2 | rs12479210 | A | DOM | | 1.225 | (0.6154 | | 2.439) | 0.5634 | |
| 2 | rs3732129 | A | DOM | | 1.223 | (0.6138 | | 2.436) | 0.5672 | |
| 2 | rs35298562 | C | DOM | | 1.218 | (0.6113 | | 2.425) | 0.5755 | |
| 2 | rs12712140 | A | DOM | | 0.86 | (0.4286 | | 1.725) | 0.6711 | |
| 2 | rs76362690 | A | DOM | | 0.86 | (0.4286 | | 1.725) | 0.6711 | |
| 2 | rs114130235 | A | DOM | | 0.882 | (0.44 | | 1.768) | 0.7234 | |
| 2 | rs10192157 | A | DOM | | 0.882 | (0.44 | | 1.768) | 0.7234 | |
| 2 | rs114130235 | G | DOM | | 1.241 | (0.322 | | 4.786) | 0.7534 | |
| 2 | rs6543119 | C | DOM | | 1.179 | (0.3818 | | 3.64) | 0.7748 | |
| 2 | rs55927292 | A | DOM | | 1.158 | (0.4102 | | 3.271) | 0.7813 | |
| 2 | rs13408661 | A | DOM | | 1.093 | (0.5486 | | 2.179) | 0.7996 | |
| 2 | rs73944273 | A | DOM | | 0.858 | (0.1754 | | 4.197) | 0.8501 | |
| 2 | rs3821204 | G | DOM | | 1.065 | (0.343 | | 3.308) | 0.9131 | |
| 2 | rs950880 | A | DOM | | 1.02 | (0.4611 | | 2.255) | 0.9618 | |
| 2 | rs1420103 | G | DOM | | 1.02 | (0.4611 | | 2.255) | 0.9618 | |
| 2 | rs3771180 | C | DOM | | 1.02 | (0.4611 | | 2.255) | 0.9618 | |
| 2 | rs1420101 | G | DOM | | 1.02 | (0.4611 | | 2.255) | 0.9618 | |
| 2 | rs12999517 | G | DOM | | 1.02 | (0.4611 | | 2.255) | 0.9618 | |
| 2 | rs10197862 | A | DOM | | 1.02 | (0.4611 | | 2.255) | 0.9618 | |
| 2 | rs4988956 | G | DOM | | 1.02 | (0.4611 | | 2.255) | 0.9618 | |
| 2 | rs873022 | A | REC | | 0.5947 | (0.2323 | | 1.522) | 0.2785 | |
| 2 | rs12479210 | A | REC | | 0.3361 | (0.04022 | | 2.808) | 0.3141 | |
| 2 | rs3732129 | A | REC | | 0.3361 | (0.04022 | | 2.808) | 0.3141 | |
| 2 | rs12905 | A | REC | | 0.7293 | (0.2995 | | 1.776) | 0.4868 | |
| 2 | rs112593736 | A | REC | | 1.509 | (0.3995 | | 5.7) | 0.544 | |
| 2 | rs35298562 | C | REC | | 0.749 | (0.1604 | | 3.497) | 0.7132 | |
| 2 | rs114130235 | A | REC | | 0.8351 | (0.2718 | | 2.566) | 0.7531 | |
| 2 | rs10192157 | A | REC | | 0.8351 | (0.2718 | | 2.566) | 0.7531 | |
| 2 | rs11123923 | A | REC | | 0.7206 | (0.08195 | | 6.335) | 0.7676 | |
| 2 | rs13011148 | A | REC | | 0.7347 | (0.0833 | | 6.48) | 0.7813 | |
| 2 | rs111533915 | C | REC | | 0.7347 | (0.0833 | | 6.48) | 0.7813 | |
| 2 | rs950880 | A | REC | | 0.8954 | (0.3812 | | 2.103) | 0.7998 | |
| 2 | rs1420103 | G | REC | | 0.8954 | (0.3812 | | 2.103) | 0.7998 | |
| 2 | rs3771180 | C | REC | | 0.8954 | (0.3812 | | 2.103) | 0.7998 | |
| 2 | rs1420101 | G | REC | | 0.8954 | (0.3812 | | 2.103) | 0.7998 | |
| 2 | rs12999517 | G | REC | | 0.8954 | (0.3812 | | 2.103) | 0.7998 | |
| 2 | rs10197862 | A | REC | | 0.8954 | (0.3812 | | 2.103) | 0.7998 | |
| 2 | rs4988956 | G | REC | | 0.8954 | (0.3812 | | 2.103) | 0.7998 | |
| 2 | rs4988956 | G | REC | | 0.8261 | (0.09239 | | 7.387) | 0.8643 | |
| 2 | rs10192036 | A | REC | | 0.8261 | (0.09239 | | 7.387) | 0.8643 | |
| 2 | rs13408661 | A | REC | | 1.109 | (0.3003 | | 4.096) | 0.8766 | |
| 2 | rs66780767 | C | REC | | 0.8448 | (0.09414 | | 7.581) | 0.8803 | |
| 2 | rs6751967 | A | REC | | 0.8448 | (0.09414 | | 7.581) | 0.8803 | |
| 2 | rs72823641 | T | REC | | 1.127 | (0.1343 | | 9.458) | 0.9123 | |
| 2 | rs17027006 | A | REC | | 1.127 | (0.1343 | | 9.458) | 0.9123 | |
| 2 | rs12469506 | A | REC | | 1.127 | (0.1343 | | 9.458) | 0.9123 | |
| 2 | rs6704565 | G | REC | | 1.127 | (0.1343 | | 9.458) | 0.9123 | |
| 2 | rs13017455 | A | REC | | 0.8928 | (0.09849 | | 8.093) | 0.9197 | |
| 2 | rs12712140 | A | REC | | 1.014 | (0.3624 | | 2.839) | 0.9783 | |
| 2 | rs76362690 | A | REC | | 1.014 | (0.3624 | | 2.839) | 0.9783 | |
| **Severe Periodontitis** | | | | | | | | | | |
| 2 | rs950880 | A | ADD | | 0.7359 | (0.5016 | | 1.079) | 0.1167 | |
| 2 | rs1420103 | G | ADD | | 0.7359 | (0.5016 | | 1.079) | 0.1167 | |
| 2 | rs3771180 | C | ADD | | 0.7359 | (0.5016 | | 1.079) | 0.1167 | |
| 2 | rs1420101 | G | ADD | | 0.7359 | (0.5016 | | 1.079) | 0.1167 | |
| 2 | rs12999517 | G | ADD | | 0.7359 | (0.5016 | | 1.079) | 0.1167 | |
| 2 | rs10197862 | A | ADD | | 0.7359 | (0.5016 | | 1.079) | 0.1167 | |
| 2 | rs4988956 | G | ADD | | 0.7359 | (0.5016 | | 1.079) | 0.1167 | |
| 2 | rs111533915 | C | ADD | | 0.705 | (0.4126 | | 1.205) | 0.2009 | |
| 2 | rs11123923 | A | ADD | | 0.7122 | (0.4173 | | 1.216) | 0.2135 | |
| 2 | rs13017455 | A | ADD | | 0.7119 | (0.4112 | | 1.233) | 0.2251 | |
| 2 | rs3821204 | G | ADD | | 0.5087 | (0.1678 | | 1.542) | 0.2323 | |
| 2 | rs13011148 | A | ADD | | 0.7298 | (0.4277 | | 1.245) | 0.2477 | |
| 2 | rs112593736 | A | ADD | | 0.7576 | (0.4697 | | 1.222) | 0.2549 | |
| 2 | rs6751967 | A | ADD | | 0.7341 | (0.4275 | | 1.26) | 0.2624 | |
| 2 | rs4988956 | G | ADD | | 0.742 | (0.4327 | | 1.272) | 0.2782 | |
| 2 | rs10192036 | A | ADD | | 0.742 | (0.4327 | | 1.272) | 0.2782 | |
| 2 | rs13408661 | A | ADD | | 0.7997 | (0.5166 | | 1.238) | 0.3161 | |
| 2 | rs66780767 | C | ADD | | 0.7607 | (0.4436 | | 1.304) | 0.3202 | |
| 2 | rs114130235 | G | ADD | | 1.558 | (0.5945 | | 4.084) | 0.367 | |
| 2 | rs73944273 | A | ADD | | 0.5512 | (0.1469 | | 2.069) | 0.3775 | |
| 2 | rs114797672 | A | ADD | | 0.6334 | (0.2011 | | 1.995) | 0.4353 | |
| 2 | rs76887186 | G | ADD | | 1.543 | (0.517 | | 4.603) | 0.437 | |
| 2 | rs17027006 | A | ADD | | 1.189 | (0.749 | | 1.888) | 0.4627 | |
| 2 | rs12999542 | C | ADD | | 1.475 | (0.5184 | | 4.199) | 0.4661 | |
| 2 | rs17026974 | T | ADD | | 1.314 | (0.6065 | | 2.847) | 0.4886 | |
| 2 | rs72823641 | T | ADD | | 1.171 | (0.7382 | | 1.857) | 0.5028 | |
| 2 | rs12469506 | A | ADD | | 1.171 | (0.7382 | | 1.857) | 0.5028 | |
| 2 | rs6704565 | G | ADD | | 1.171 | (0.7382 | | 1.857) | 0.5028 | |
| 2 | rs3771177 | A | ADD | | 0.8398 | (0.497 | | 1.419) | 0.5143 | |
| 2 | rs1420101 | A | ADD | | 1.353 | (0.4609 | | 3.971) | 0.5823 | |
| 2 | rs148548829 | C | ADD | | 1.115 | (0.7108 | | 1.748) | 0.6361 | |
| 2 | rs13019081 | A | ADD | | 0.8861 | (0.5297 | | 1.482) | 0.6451 | |
| 2 | rs11693204 | A | ADD | | 1.127 | (0.6476 | | 1.962) | 0.6719 | |
| 2 | rs55927292 | A | ADD | | 0.8359 | (0.3494 | | 2) | 0.6872 | |
| 2 | rs10206753 | G | ADD | | 1.081 | (0.6872 | | 1.699) | 0.737 | |
| 2 | rs873022 | A | ADD | | 1.053 | (0.727 | | 1.525) | 0.7849 | |
| 2 | rs12905 | A | ADD | | 1.05 | (0.7254 | | 1.521) | 0.7951 | |
| 2 | rs35298562 | C | ADD | | 0.9568 | (0.6257 | | 1.463) | 0.8383 | |
| 2 | rs12479210 | A | ADD | | 0.9671 | (0.6329 | | 1.478) | 0.877 | |
| 2 | rs12712140 | A | ADD | | 0.9729 | (0.6569 | | 1.441) | 0.891 | |
| 2 | rs76362690 | A | ADD | | 0.9729 | (0.6569 | | 1.441) | 0.891 | |
| 2 | rs6543119 | C | ADD | | 1.03 | (0.4254 | | 2.492) | 0.9483 | |
| 2 | rs3732129 | A | ADD | | 0.9891 | (0.6478 | | 1.51) | 0.9596 | |
| 2 | rs114130235 | A | ADD | | 0.9929 | (0.6703 | | 1.471) | 0.9715 | |
| 2 | rs10192157 | A | ADD | | 0.9929 | (0.6703 | | 1.471) | 0.9715 | |
| 2 | rs73944273 | A | DOM | | 0.5512 | (0.1469 | | 2.069) | 0.3775 | |
| 2 | rs13011148 | A | DOM | | 0.7587 | (0.4119 | | 1.397) | 0.3755 | |
| 2 | rs950880 | A | DOM | | 0.6365 | (0.3614 | | 1.121) | 0.1177 | |
| 2 | rs11693204 | A | DOM | | 1.077 | (0.5796 | | 2) | 0.8153 | |
| 2 | rs72823641 | T | DOM | | 1.083 | (0.6329 | | 1.854) | 0.7705 | |
| 2 | rs66780767 | C | DOM | | 0.7902 | (0.4282 | | 1.458) | 0.4513 | |
| 2 | rs1420103 | G | DOM | | 0.6365 | (0.3614 | | 1.121) | 0.1177 | |
| 2 | rs12479210 | A | DOM | | 1.041 | (0.6179 | | 1.755) | 0.8789 | |
| 2 | rs13019081 | A | DOM | | 0.9645 | (0.5354 | | 1.738) | 0.9042 | |
| 2 | rs12712140 | A | DOM | | 1.078 | (0.6294 | | 1.846) | 0.7845 | |
| 2 | rs76362690 | A | DOM | | 1.078 | (0.6294 | | 1.846) | 0.7845 | |
| 2 | rs112593736 | A | DOM | | 0.7632 | (0.4368 | | 1.334) | 0.3427 | |
| 2 | rs17026974 | T | DOM | | 1.211 | (0.5124 | | 2.863) | 0.6624 | |
| 2 | rs3771180 | C | DOM | | 0.6365 | (0.3614 | | 1.121) | 0.1177 | |
| 2 | rs13408661 | A | DOM | | 0.8393 | (0.4962 | | 1.42) | 0.5135 | |
| 2 | rs873022 | A | DOM | | 1.094 | (0.6034 | | 1.985) | 0.7666 | |
| 2 | rs3771177 | A | DOM | | 0.8084 | (0.4458 | | 1.466) | 0.4836 | |
| 2 | rs114797672 | A | DOM | | 0.6334 | (0.2011 | | 1.995) | 0.4353 | |
| 2 | rs3732129 | A | DOM | | 1.078 | (0.6393 | | 1.818) | 0.7784 | |
| 2 | rs1420101 | A | DOM | | 1.353 | (0.4609 | | 3.971) | 0.5823 | |
| 2 | rs1420101 | G | DOM | | 0.6365 | (0.3614 | | 1.121) | 0.1177 | |
| 2 | rs12999517 | G | DOM | | 0.6365 | (0.3614 | | 1.121) | 0.1177 | |
| 2 | rs35298562 | C | DOM | | 1.025 | (0.6081 | | 1.729) | 0.9254 | |
| 2 | rs12905 | A | DOM | | 1.094 | (0.6034 | | 1.985) | 0.7666 | |
| 2 | rs3821204 | G | DOM | | 0.5087 | (0.1678 | | 1.542) | 0.2323 | |
| 2 | rs6543119 | C | DOM | | 1.051 | (0.4263 | | 2.592) | 0.9137 | |
| 2 | rs13017455 | A | DOM | | 0.7252 | (0.3896 | | 1.35) | 0.311 | |
| 2 | rs55927292 | A | DOM | | 0.8516 | (0.3487 | | 2.08) | 0.7243 | |
| 2 | rs17027006 | A | DOM | | 1.104 | (0.6438 | | 1.894) | 0.7188 | |
| 2 | rs12999542 | C | DOM | | 1.475 | (0.5184 | | 4.199) | 0.4661 | |
| 2 | rs12469506 | A | DOM | | 1.083 | (0.6329 | | 1.854) | 0.7705 | |
| 2 | rs10197862 | A | DOM | | 0.6365 | (0.3614 | | 1.121) | 0.1177 | |
| 2 | rs111533915 | C | DOM | | 0.7253 | (0.3942 | | 1.335) | 0.302 | |
| 2 | rs148548829 | C | DOM | | 1.139 | (0.6608 | | 1.962) | 0.6401 | |
| 2 | rs6751967 | A | DOM | | 0.7548 | (0.4095 | | 1.391) | 0.3673 | |
| 2 | rs6704565 | G | DOM | | 1.083 | (0.6329 | | 1.854) | 0.7705 | |
| 2 | rs76887186 | G | DOM | | 1.543 | (0.517 | | 4.603) | 0.437 | |
| 2 | rs11123923 | A | DOM | | 0.7361 | (0.3997 | | 1.356) | 0.3255 | |
| 2 | rs4988956 | G | DOM | | 0.7665 | (0.4155 | | 1.414) | 0.3945 | |
| 2 | rs4988956 | G | DOM | | 0.6365 | (0.3614 | | 1.121) | 0.1177 | |
| 2 | rs114130235 | A | DOM | | 1.105 | (0.6457 | | 1.89) | 0.716 | |
| 2 | rs114130235 | G | DOM | | 1.558 | (0.5945 | | 4.084) | 0.367 | |
| 2 | rs10192036 | A | DOM | | 0.7665 | (0.4155 | | 1.414) | 0.3945 | |
| 2 | rs10192157 | A | DOM | | 1.105 | (0.6457 | | 1.89) | 0.716 | |
| 2 | rs10206753 | G | DOM | | 1.088 | (0.6294 | | 1.882) | 0.7621 | |
| 2 | rs17027006 | A | REC | | 2.213 | (0.6564 | | 7.461) | 0.2002 | |
| 2 | rs72823641 | T | REC | | 2.196 | (0.6515 | | 7.399) | 0.2045 | |
| 2 | rs12469506 | A | REC | | 2.196 | (0.6515 | | 7.399) | 0.2045 | |
| 2 | rs6704565 | G | REC | | 2.196 | (0.6515 | | 7.399) | 0.2045 | |
| 2 | rs17026974 | T | REC | | 4.88 | (0.3724 | | 63.96) | 0.2273 | |
| 2 | rs11123923 | A | REC | | 0.2631 | (0.02785 | | 2.487) | 0.244 | |
| 2 | rs13408661 | A | REC | | 0.4643 | (0.1266 | | 1.702) | 0.2471 | |
| 2 | rs13011148 | A | REC | | 0.267 | (0.02816 | | 2.532) | 0.2499 | |
| 2 | rs111533915 | C | REC | | 0.267 | (0.02816 | | 2.532) | 0.2499 | |
| 2 | rs13019081 | A | REC | | 0.2974 | (0.03094 | | 2.86) | 0.2937 | |
| 2 | rs4988956 | G | REC | | 0.2974 | (0.03094 | | 2.86) | 0.2937 | |
| 2 | rs10192036 | A | REC | | 0.2974 | (0.03094 | | 2.86) | 0.2937 | |
| 2 | rs66780767 | C | REC | | 0.3025 | (0.03133 | | 2.92) | 0.3013 | |
| 2 | rs6751967 | A | REC | | 0.3025 | (0.03133 | | 2.92) | 0.3013 | |
| 2 | rs13017455 | A | REC | | 0.3039 | (0.03144 | | 2.938) | 0.3035 | |
| 2 | rs112593736 | A | REC | | 0.4628 | (0.09548 | | 2.244) | 0.3388 | |
| 2 | rs950880 | A | REC | | 0.7202 | (0.3675 | | 1.412) | 0.3391 | |
| 2 | rs1420103 | G | REC | | 0.7202 | (0.3675 | | 1.412) | 0.3391 | |
| 2 | rs3771180 | C | REC | | 0.7202 | (0.3675 | | 1.412) | 0.3391 | |
| 2 | rs1420101 | G | REC | | 0.7202 | (0.3675 | | 1.412) | 0.3391 | |
| 2 | rs12999517 | G | REC | | 0.7202 | (0.3675 | | 1.412) | 0.3391 | |
| 2 | rs10197862 | A | REC | | 0.7202 | (0.3675 | | 1.412) | 0.3391 | |
| 2 | rs4988956 | G | REC | | 0.7202 | (0.3675 | | 1.412) | 0.3391 | |
| 2 | rs11693204 | A | REC | | 2.129 | (0.3235 | | 14.02) | 0.4318 | |
| 2 | rs35298562 | C | REC | | 0.6666 | (0.21 | | 2.116) | 0.4914 | |
| 2 | rs12479210 | A | REC | | 0.668 | (0.2104 | | 2.121) | 0.4937 | |
| 2 | rs3732129 | A | REC | | 0.668 | (0.2104 | | 2.121) | 0.4937 | |
| 2 | rs12712140 | A | REC | | 0.7461 | (0.3209 | | 1.735) | 0.4962 | |
| 2 | rs76362690 | A | REC | | 0.7461 | (0.3209 | | 1.735) | 0.4962 | |
| 2 | rs114130235 | A | REC | | 0.766 | (0.3285 | | 1.786) | 0.5372 | |
| 2 | rs10192157 | A | REC | | 0.766 | (0.3285 | | 1.786) | 0.5372 | |
| 2 | rs148548829 | C | REC | | 1.159 | (0.3369 | | 3.986) | 0.8151 | |
| 2 | rs10206753 | G | REC | | 1.159 | (0.3369 | | 3.986) | 0.8151 | |
| 2 | rs873022 | A | REC | | 1.047 | (0.5637 | | 1.944) | 0.8848 | |
| 2 | rs12905 | A | REC | | 1.04 | (0.5599 | | 1.93) | 0.9023 | |
| 2 | rs3771177 | A | REC | | 0.9001 | (0.1668 | | 4.858) | 0.9026 | |
|  | | | | | |  |  | | |  |
| ***IL33* gene** | | | | | | | | | | |
| **Periodontitis** | | | | | | | | | | |
| \| **CHR** \| **SNV** \| **Variant allele A1** \| **Model** \| **OR_Adjusted_ (95%CI)** \| **p-value** \| \| --- \| --- \| --- \| --- \| --- \| --- \| | | | | | | | | | | |
| 9 | rs72614080 | A | | ADD | 1.418 | (0.7309 | | 2.75) | 0.3018 | |
| 9 | rs2066362 | A | | ADD | 0.8408 | (0.5868 | | 1.205) | 0.3446 | |
| 9 | rs1891385 | C | | ADD | 1.211 | (0.6821 | | 2.151) | 0.513 | |
| 9 | rs118148121 | T | | ADD | 1.922 | (0.6685 | | 5.528) | 0.2253 | |
| 9 | rs10435816 | G | | ADD | 1.146 | (0.8316 | | 1.578) | 0.4058 | |
| 9 | rs12551256 | G | | ADD | 0.9304 | (0.6603 | | 1.311) | 0.6801 | |
| 9 | rs7025417 | G | | ADD | 1.018 | (0.7017 | | 1.478) | 0.9241 | |
| 9 | rs78100995 | C | | ADD | 0.8455 | (0.4938 | | 1.448) | 0.5409 | |
| 9 | rs1330383 | A | | ADD | 1.014 | (0.7308 | | 1.406) | 0.9345 | |
| 9 | rs10975519 | A | | ADD | 0.9847 | (0.7098 | | 1.366) | 0.9267 | |
| 9 | rs142772030 | A | | ADD | 0.6907 | (0.1382 | | 3.453) | 0.6522 | |
| 9 | rs16924241 | G | | ADD | 1.526 | (0.5457 | | 4.268) | 0.4204 | |
| 9 | rs1048274 | A | | ADD | 0.9795 | (0.704 | | 1.363) | 0.9019 | |
| 9 | rs16924243 | G | | ADD | 0.863 | (0.5225 | | 1.426) | 0.5652 | |
| 9 | rs72614080 | A | | DOM | 1.418 | (0.7309 | | 2.75) | 0.3018 | |
| 9 | rs2066362 | A | | DOM | 0.7343 | (0.4674 | | 1.154) | 0.1801 | |
| 9 | rs1891385 | C | | DOM | 1.236 | (0.675 | | 2.265) | 0.4919 | |
| 9 | rs118148121 | T | DOM | | 1.922 | (0.6685 | | 5.528) | 0.2253 | |
| 9 | rs10435816 | G | DOM | | 1.079 | (0.6727 | | 1.731) | 0.7524 | |
| 9 | rs12551256 | G | DOM | | 0.8538 | (0.5445 | | 1.339) | 0.4911 | |
| 9 | rs7025417 | G | DOM | | 1.014 | (0.6371 | | 1.614) | 0.9534 | |
| 9 | rs78100995 | C | DOM | | 0.7771 | (0.4303 | | 1.404) | 0.4032 | |
| 9 | rs1330383 | A | DOM | | 0.9944 | (0.6322 | | 1.564) | 0.9808 | |
| 9 | rs10975519 | A | DOM | | 1.051 | (0.6669 | | 1.655) | 0.831 | |
| 9 | rs142772030 | A | DOM | | 0.6907 | (0.1382 | | 3.453) | 0.6522 | |
| 9 | rs16924241 | G | DOM | | 1.67 | (0.5543 | | 5.032) | 0.362 | |
| 9 | rs1048274 | A | DOM | | 1.015 | (0.6443 | | 1.599) | 0.9484 | |
| 9 | rs16924243 | G | DOM | | 0.8947 | (0.5127 | | 1.561) | 0.6953 | |
| 9 | rs2066362 | A | REC | | 1.104 | (0.497 | | 2.451) | 0.8086 | |
| 9 | rs1891385 | C | REC | | 1.045 | (0.05557 | | 19.64) | 0.9767 | |
| 9 | rs10435816 | G | REC | | 1.395 | (0.7866 | | 2.475) | 0.2547 | |
| 9 | rs12551256 | G | REC | | 1.092 | (0.5264 | | 2.266) | 0.8128 | |
| 9 | rs7025417 | G | REC | | 1.062 | (0.4116 | | 2.739) | 0.9013 | |
| 9 | rs78100995 | C | REC | | 1.944 | (0.2482 | | 15.22) | 0.5268 | |
| 9 | rs1330383 | A | REC | | 1.073 | (0.5492 | | 2.095) | 0.8372 | |
| 9 | rs10975519 | A | REC | | 0.8377 | (0.4206 | | 1.668) | 0.6143 | |
| 9 | rs1048274 | A | REC | | 0.8841 | (0.4432 | | 1.763) | 0.7266 | |
| 9 | rs16924243 | G | REC | | 0.417 | (0.04717 | | 3.686) | 0.4314 | |
|  |  |  |  | |  |  | |  |  | |
| **Moderate Periodontitis** | | | | | | | | | | |
| 9 | rs72614080 | A | ADD | | 0.8716 | (0.2795 | | 2.717) | 0.8127 | |
| 9 | rs2066362 | A | ADD | | 0.5935 | (0.3268 | | 1.078) | 0.08658 | |
| 9 | rs1891385 | C | ADD | | 0.3215 | (0.07203 | | 1.435) | 0.137 | |
| 9 | rs118148121 | T | ADD | | 2.315 | (0.4343 | | 12.34) | 0.3255 | |
| 9 | rs10435816 | G | ADD | | 1.487 | (0.9028 | | 2.448) | 0.1192 | |
| 9 | rs12551256 | G | ADD | | 0.9182 | (0.5292 | | 1.593) | 0.7616 | |
| 9 | rs7025417 | G | ADD | | 1.094 | (0.6078 | | 1.971) | 0.7637 | |
| 9 | rs78100995 | C | ADD | | 1.261 | (0.5814 | | 2.736) | 0.557 | |
| 9 | rs1330383 | A | ADD | | 0.9152 | (0.5458 | | 1.535) | 0.7367 | |
| 9 | rs10975519 | A | ADD | | 0.9509 | (0.574 | | 1.575) | 0.845 | |
| 9 | rs142772030 | A | ADD | | 1.057 | (0.1231 | | 9.077) | 0.9597 | |
| 9 | rs16924241 | G | ADD | | 2.374 | (0.6732 | | 8.371) | 0.1787 | |
| 9 | rs1048274 | A | ADD | | 0.9476 | (0.5686 | | 1.579) | 0.8363 | |
| 9 | rs16924243 | G | ADD | | 0.8688 | (0.401 | | 1.882) | 0.7214 | |
| 9 | rs72614080 | A | DOM | | 0.8716 | (0.2795 | | 2.717) | 0.8127 | |
| 9 | rs1891385 | C | DOM | | 0.3184 | (0.0703 | | 1.442) | 0.1376 | |
| 9 | rs118148121 | T | DOM | | 2.315 | (0.4343 | | 12.34) | 0.3255 | |
| 9 | rs10435816 | G | DOM | | 1.337 | (0.6354 | | 2.815) | 0.4439 | |
| 9 | rs12551256 | G | DOM | | 0.9026 | (0.4521 | | 1.802) | 0.7713 | |
| 9 | rs7025417 | G | DOM | | 1.102 | (0.539 | | 2.253) | 0.7903 | |
| 9 | rs78100995 | C | DOM | | 1.145 | (0.488 | | 2.687) | 0.7556 | |
| 9 | rs1330383 | A | DOM | | 0.8086 | (0.4022 | | 1.626) | 0.5512 | |
| 9 | rs10975519 | A | DOM | | 0.9134 | (0.4531 | | 1.841) | 0.8001 | |
| 9 | rs142772030 | A | DOM | | 1.057 | (0.1231 | | 9.077) | 0.9597 | |
| 9 | rs16924241 | G | DOM | | 2.984 | (0.735 | | 12.11) | 0.1262 | |
| 9 | rs1048274 | A | DOM | | 0.8863 | (0.4397 | | 1.786) | 0.7357 | |
| 9 | rs16924243 | G | DOM | | 0.9582 | (0.4105 | | 2.237) | 0.9214 | |
| 9 | rs2066362 | A | REC | | 0.9431 | (0.2616 | | 3.4) | 0.9286 | |
| 9 | rs10435816 | G | REC | | 2.228 | (0.9808 | | 5.06) | 0.05566 | |
| 9 | rs12551256 | G | REC | | 0.8949 | (0.2483 | | 3.225) | 0.8651 | |
| 9 | rs7025417 | G | REC | | 1.185 | (0.2539 | | 5.529) | 0.8291 | |
| 9 | rs78100995 | C | REC | | 5.139 | (0.4116 | | 64.15) | 0.2038 | |
| 9 | rs1330383 | A | REC | | 1.113 | (0.396 | | 3.13) | 0.8388 | |
| 9 | rs10975519 | A | REC | | 0.984 | (0.3517 | | 2.753) | 0.9755 | |
| 9 | rs1048274 | A | REC | | 1.04 | (0.3712 | | 2.915) | 0.94 | |
| **Severe Periodontitis** | | | | | | | | | | |
| 9 | rs72614080 | A | ADD | | 1.708 | (0.8207 | | 3.553) | 0.1523 | |
| 9 | rs2066362 | A | ADD | | 0.918 | (0.6048 | | 1.393) | 0.6879 | |
| 9 | rs1891385 | C | ADD | | 1.731 | (0.9372 | | 3.197) | 0.07965 | |
| 9 | rs118148121 | T | ADD | | 2.124 | (0.6446 | | 7) | 0.2156 | |
| 9 | rs10435816 | G | ADD | | 1.029 | (0.7039 | | 1.506) | 0.8809 | |
| 9 | rs12551256 | G | ADD | | 0.9551 | (0.6425 | | 1.42) | 0.8205 | |
| 9 | rs7025417 | G | ADD | | 0.999 | (0.6488 | | 1.538) | 0.9964 | |
| 9 | rs78100995 | C | ADD | | 0.6949 | (0.3554 | | 1.359) | 0.2873 | |
| 9 | rs1330383 | A | ADD | | 1.079 | (0.7391 | | 1.576) | 0.6924 | |
| 9 | rs10975519 | A | ADD | | 1.007 | (0.6888 | | 1.473) | 0.9699 | |
| 9 | rs142772030 | A | ADD | | 0.478 | (0.05523 | | 4.137) | 0.5026 | |
| 9 | rs16924241 | G | ADD | | 1.04 | (0.2714 | | 3.986) | 0.9541 | |
| 9 | rs1048274 | A | ADD | | 1.003 | (0.6828 | | 1.472) | 0.9898 | |
| 9 | rs16924243 | G | ADD | | 0.8228 | (0.4546 | | 1.489) | 0.5192 | |
| 9 | rs72614080 | A | DOM | | 1.418 | (0.7309 | | 2.75) | 0.3018 | |
| 9 | rs2066362 | A | DOM | | 0.7343 | (0.4674 | | 1.154) | 0.1801 | |
| 9 | rs1891385 | C | DOM | | 1.236 | (0.675 | | 2.265) | 0.4919 | |
| 9 | rs118148121 | T | DOM | | 1.922 | (0.6685 | | 5.528) | 0.2253 | |
| 9 | rs10435816 | G | DOM | | 1.079 | (0.6727 | | 1.731) | 0.7524 | |
| 9 | rs12551256 | G | DOM | | 0.8538 | (0.5445 | | 1.339) | 0.4911 | |
| 9 | rs7025417 | G | DOM | | 1.014 | (0.6371 | | 1.614) | 0.9534 | |
| 9 | rs78100995 | C | DOM | | 0.7771 | (0.4303 | | 1.404) | 0.4032 | |
| 9 | rs2066362 | A | DOM | | 0.7343 | (0.4674 | | 1.154) | 0.1801 | |
| 9 | rs1891385 | C | DOM | | 1.236 | (0.675 | | 2.265) | 0.4919 | |
| 9 | rs118148121 | T | DOM | | 1.922 | (0.6685 | | 5.528) | 0.2253 | |
| 9 | rs10435816 | G | DOM | | 1.079 | (0.6727 | | 1.731) | 0.7524 | |
| 9 | rs12551256 | G | DOM | | 0.8538 | (0.5445 | | 1.339) | 0.4911 | |
| 9 | rs7025417 | G | DOM | | 1.014 | (0.6371 | | 1.614) | 0.9534 | |
| 9 | rs78100995 | C | DOM | | 0.7771 | (0.4303 | | 1.404) | 0.4032 | |
| 9 | rs1330383 | A | DOM | | 0.9944 | (0.6322 | | 1.564) | 0.9808 | |
| 9 | rs10975519 | A | DOM | | 1.051 | (0.6669 | | 1.655) | 0.831 | |
| 9 | rs142772030 | A | DOM | | 0.6907 | (0.1382 | | 3.453) | 0.6522 | |
| 9 | rs16924241 | G | DOM | | 1.67 | (0.5543 | | 5.032) | 0.362 | |
| 9 | rs1048274 | A | DOM | | 1.015 | (0.6443 | | 1.599) | 0.9484 | |
| 9 | rs16924243 | G | DOM | | 0.8947 | (0.5127 | | 1.561) | 0.6953 | |
| 9 | rs2066362 | A | REC | | 1.104 | (0.4408 | | 2.764) | 0.833 | |
| 9 | rs1891385 | C | REC | | 1.578 | (0.08292 | | 30.02) | 0.7616 | |
| 9 | rs10435816 | G | REC | | 1.102 | (0.5457 | | 2.224) | 0.7871 | |
| 9 | rs12551256 | G | REC | | 1.218 | (0.5399 | | 2.747) | 0.6349 | |
| 9 | rs7025417 | G | REC | | 1.037 | (0.3534 | | 3.042) | 0.9474 | |
| 9 | rs78100995 | C | REC | | 1.259 | (0.1014 | | 15.63) | 0.8578 | |
| 9 | rs1330383 | A | REC | | 1.044 | (0.4822 | | 2.262) | 0.9123 | |
| 9 | rs10975519 | A | REC | | 0.7452 | (0.3279 | | 1.694) | 0.4826 | |
| 9 | rs1048274 | A | REC | | 0.7893 | (0.3468 | | 1.796) | 0.5728 | |
| 9 | rs16924243 | G | REC | | 0.6418 | (0.06972 | | 5.909) | 0.6954 | |

CHR: Chromosome; OR_Adjusted_: odds ratio adjusted for total years of education, diagnosis of asthma, flossing, age, body mass index (BMI), mouth breathing habit, and PC1; 95%CI: confidence interval of 95%; p: statistical significance level (p≤0.05).
