# Supplementary material for: Single nucleotide variants in the IL33 and IL1RL1 (ST2) genes are associated with periodontitis and with Aggregatibacter actinomycetemcomitans in the dental plaque biofilm: A putative role in understanding the host immune response in periodontitis
Source: PLoS One. 2023 Mar 22;18(3):e0283179. doi: 10.1371/journal.pone.0283179 (PMC10032506; doi:10.1371/journal.pone.0283179)
Supplement: S3 Table — (DOCX) [file pone.0283179.s003.docx]

**S3 Table.** Adjusted association measurements, odds ratio and 95% confidence interval, between the SNVs of the ST2 gene and IL33 gene, and Aggregatibacter actinomycetemcomitans relative amount, considering additive (ADD), dominant (DOM) and recessive (REC) genetic model

| ***ST2* gene** | | | | | | | |
| --- | --- | --- | --- | --- | --- | --- | --- |
| \| ***Aggregatibacter actinomycetemcomitans*** \| \| --- \| | | | | | | | |
| **CHR** | **SNV** | **Variant allele A1** | **Model** | **OR_Adjusted_ (95%CI)** | | | **p-value** |
| 2 | rs73944273 | A | ADD | 0.588 | (0.1138 | 3.037) | 0.5261 |
| 2 | rs13011148 | A | ADD | 0.5309 | (0.2921 | 0.9649) | 0.03778 |
| 2 | rs950880 | A | ADD | 0.8548 | (0.5808 | 1.258) | 0.4263 |
| 2 | rs11693204 | A | ADD | 1.427 | (0.781 | 2.609) | 0.2474 |
| 2 | rs72823641 | T | ADD | 1.116 | (0.6671 | 1.865) | 0.6768 |
| 2 | rs66780767 | C | ADD | 0.5481 | (0.3004 | 1) | 0.05006 |
| 2 | rs1420103 | G | ADD | 0.8548 | (0.5808 | 1.258) | 0.4263 |
| 2 | rs12479210 | A | ADD | 0.7834 | (0.4857 | 1.264) | 0.3169 |
| 2 | rs13019081 | A | ADD | 0.526 | (0.2849 | 0.9711) | 0.04 |
| 2 | rs12712140 | A | ADD | 0.7465 | (0.4893 | 1.139) | 0.1749 |
| 2 | rs76362690 | A | ADD | 0.7465 | (0.4893 | 1.139) | 0.1749 |
| 2 | rs112593736 | A | ADD | 0.5897 | (0.3525 | 0.9864) | 0.0442 |
| 2 | rs17026974 | T | ADD | 1.69 | (0.7049 | 4.052) | 0.2395 |
| 2 | rs17639215 | A | ADD | 0.6693 | (0.06673 | 6.713) | 0.7329 |
| 2 | rs3771180 | C | ADD | 0.8548 | (0.5808 | 1.258) | 0.4263 |
| 2 | rs13408661 | A | ADD | 0.7337 | (0.4585 | 1.174) | 0.1967 |
| 2 | rs873022 | A | ADD | 0.8717 | (0.5794 | 1.311) | 0.5097 |
| 2 | rs3771177 | A | ADD | 1.149 | (0.6784 | 1.945) | 0.6059 |
| 2 | rs114797672 | A | ADD | 0.9228 | (0.3499 | 2.434) | 0.871 |
| 2 | rs3732129 | A | ADD | 0.7564 | (0.4678 | 1.223) | 0.2548 |
| 2 | rs1420101 | A | ADD | 0.6934 | (0.2091 | 2.299) | 0.5494 |
| 2 | rs1420101 | G | ADD | 0.8548 | (0.5808 | 1.258) | 0.4263 |
| 2 | rs12999517 | G | ADD | 0.8548 | (0.5808 | 1.258) | 0.4263 |
| 2 | rs35298562 | C | ADD | 0.8157 | (0.5101 | 1.304) | 0.395 |
| 2 | rs12905 | A | ADD | 0.9184 | (0.6145 | 1.373) | 0.6779 |
| 2 | rs3771175 | A | ADD | 0.6693 | (0.06673 | 6.713) | 0.7329 |
| 2 | rs3821204 | G | ADD | 1.261 | (0.5691 | 2.795) | 0.5675 |
| 2 | rs6543119 | C | ADD | 1.042 | (0.4145 | 2.621) | 0.9299 |
| 2 | rs13017455 | A | ADD | 0.6011 | (0.3339 | 1.082) | 0.0897 |
| 2 | rs55927292 | A | ADD | 1.359 | (0.6867 | 2.69) | 0.3784 |
| 2 | rs17027006 | A | ADD | 1.116 | (0.6671 | 1.865) | 0.6768 |
| 2 | rs12999542 | C | ADD | 1.085 | (0.302 | 3.9) | 0.9002 |
| 2 | rs12469506 | A | ADD | 1.116 | (0.6671 | 1.865) | 0.6768 |
| 2 | rs10197862 | A | ADD | 0.8548 | (0.5808 | 1.258) | 0.4263 |
| 2 | rs111533915 | C | ADD | 0.5033 | (0.2763 | 0.9167) | 0.02481 |
| 2 | rs148548829 | C | ADD | 1.185 | (0.7356 | 1.909) | 0.4855 |
| 2 | rs6751967 | A | ADD | 0.5191 | (0.284 | 0.949) | 0.03317 |
| 2 | rs6704565 | G | ADD | 1.116 | (0.6671 | 1.865) | 0.6768 |
| 2 | rs76887186 | G | ADD | 1.019 | (0.2896 | 3.585) | 0.9768 |
| 2 | rs11123923 | A | ADD | 0.5663 | (0.3157 | 1.016) | 0.05657 |
| 2 | rs4988956 | G | ADD | 0.5851 | (0.3248 | 1.054) | 0.07432 |
| 2 | rs4988956 | G | ADD | 0.8548 | (0.5808 | 1.258) | 0.4263 |
| 2 | rs114130235 | A | ADD | 0.8033 | (0.5265 | 1.226) | 0.3095 |
| 2 | rs114130235 | G | ADD | 1.353 | (0.4483 | 4.082) | 0.5918 |
| 2 | rs10192036 | A | ADD | 0.5851 | (0.3248 | 1.054) | 0.07432 |
| 2 | rs10192157 | A | ADD | 0.8033 | (0.5265 | 1.226) | 0.3095 |
| 2 | rs73944273 | A | DOM | 0.588 | (0.1138 | 3.037) | 0.5261 |
| 2 | rs950880 | A | DOM | 0.7728 | (0.4293 | 1.391) | 0.39 |
| 2 | rs11693204 | A | DOM | 1.37 | (0.7039 | 2.666) | 0.3541 |
| 2 | rs72823641 | T | DOM | 1.053 | (0.5948 | 1.865) | 0.8589 |
| 2 | rs1420103 | G | DOM | 0.7728 | (0.4293 | 1.391) | 0.39 |
| 2 | rs12479210 | A | DOM | 0.7472 | (0.4317 | 1.293) | 0.2978 |
| 2 | rs12712140 | A | DOM | 0.5937 | (0.3439 | 1.025) | 0.06122 |
| 2 | rs76362690 | A | DOM | 0.5937 | (0.3439 | 1.025) | 0.06122 |
| 2 | rs17026974 | T | DOM | 1.603 | (0.6288 | 4.088) | 0.3229 |
| 2 | rs17639215 | A | DOM | 0.6693 | (0.06673 | 6.713) | 0.7329 |
| 2 | rs3771180 | C | DOM | 0.7728 | (0.4293 | 1.391) | 0.39 |
| 2 | rs13408661 | A | DOM | 0.6582 | (0.3787 | 1.144) | 0.1379 |
| 2 | rs873022 | A | DOM | 0.7057 | (0.3946 | 1.262) | 0.2399 |
| 2 | rs3771177 | A | DOM | 1.054 | (0.5841 | 1.903) | 0.8609 |
| 2 | rs114797672 | A | DOM | 0.9228 | (0.3499 | 2.434) | 0.871 |
| 2 | rs3732129 | A | DOM | 0.713 | (0.4109 | 1.237) | 0.229 |
| 2 | rs1420101 | A | DOM | 0.6934 | (0.2091 | 2.299) | 0.5494 |
| 2 | rs1420101 | G | DOM | 0.7728 | (0.4293 | 1.391) | 0.39 |
| 2 | rs12999517 | G | DOM | 0.7728 | (0.4293 | 1.391) | 0.39 |
| 2 | rs35298562 | C | DOM | 0.7471 | (0.4316 | 1.293) | 0.2975 |
| 2 | rs12905 | A | DOM | 0.7057 | (0.3946 | 1.262) | 0.2399 |
| 2 | rs3771175 | A | DOM | 0.6693 | (0.06673 | 6.713) | 0.7329 |
| 2 | rs3821204 | G | DOM | 1.261 | (0.5691 | 2.795) | 0.5675 |
| 2 | rs6543119 | C | DOM | 1.042 | (0.4145 | 2.621) | 0.9299 |
| 2 | rs13017455 | A | DOM | 0.5208 | (0.2636 | 1.029) | 0.06047 |
| 2 | rs55927292 | A | DOM | 1.185 | (0.5627 | 2.497) | 0.6546 |
| 2 | rs17027006 | A | DOM | 1.053 | (0.5948 | 1.865) | 0.8589 |
| 2 | rs12999542 | C | DOM | 1.085 | (0.302 | 3.9) | 0.9002 |
| 2 | rs12469506 | A | DOM | 1.053 | (0.5948 | 1.865) | 0.8589 |
| 2 | rs10197862 | A | DOM | 0.7728 | (0.4293 | 1.391) | 0.39 |
| 2 | rs148548829 | C | DOM | 1.033 | (0.5783 | 1.844) | 0.9134 |
| 2 | rs6704565 | G | DOM | 1.053 | (0.5948 | 1.865) | 0.8589 |
| 2 | rs76887186 | G | DOM | 1.019 | (0.2896 | 3.585) | 0.9768 |
| 2 | rs4988956 | G | DOM | 0.7728 | (0.4293 | 1.391) | 0.39 |
| 2 | rs114130235 | A | DOM | 0.657 | (0.3809 | 1.133) | 0.1309 |
| 2 | rs114130235 | G | DOM | 1.353 | (0.4483 | 4.082) | 0.5918 |
| 2 | rs10192157 | A | DOM | 0.657 | (0.3809 | 1.133) | 0.1309 |
| 2 | rs10206753 | G | DOM | 1.072 | (0.5992 | 1.917) | 0.8152 |
| 2 | rs13011148 | A | REC | 0.6501 | (0.1266 | 3.337) | 0.6058 |
| 2 | rs950880 | A | REC | 0.8676 | (0.4427 | 1.7) | 0.6792 |
| 2 | rs11693204 | A | REC | 4.312 | (0.3652 | 50.92) | 0.246 |
| 2 | rs72823641 | T | REC | 2.288 | (0.3662 | 14.3) | 0.3759 |
| 2 | rs66780767 | C | REC | 0.7369 | (0.1388 | 3.912) | 0.72 |
| 2 | rs1420103 | G | REC | 0.8676 | (0.4427 | 1.7) | 0.6792 |
| 2 | rs12479210 | A | REC | 0.8071 | (0.2011 | 3.239) | 0.7625 |
| 2 | rs13019081 | A | REC | 0.3817 | (0.0435 | 3.349) | 0.3847 |
| 2 | rs12712140 | A | REC | 1.059 | (0.442 | 2.537) | 0.8979 |
| 2 | rs76362690 | A | REC | 1.059 | (0.442 | 2.537) | 0.8979 |
| 2 | rs112593736 | A | REC | 0.7071 | (0.1803 | 2.773) | 0.6191 |
| 2 | rs3771180 | C | REC | 0.8676 | (0.4427 | 1.7) | 0.6792 |
| 2 | rs13408661 | A | REC | 0.9183 | (0.2658 | 3.172) | 0.8928 |
| 2 | rs873022 | A | REC | 1.11 | (0.5345 | 2.304) | 0.7799 |
| 2 | rs3771177 | A | REC | 3.139 | (0.4994 | 19.73) | 0.2226 |
| 2 | rs3732129 | A | REC | 0.8071 | (0.2011 | 3.239) | 0.7625 |
| 2 | rs1420101 | G | REC | 0.8676 | (0.4427 | 1.7) | 0.6792 |
| 2 | rs12999517 | G | REC | 0.8676 | (0.4427 | 1.7) | 0.6792 |
| 2 | rs35298562 | C | REC | 1.06 | (0.2981 | 3.77) | 0.9281 |
| 2 | rs12905 | A | REC | 1.285 | (0.6335 | 2.608) | 0.4866 |
| 2 | rs13017455 | A | REC | 0.7369 | (0.1388 | 3.912) | 0.72 |
| 2 | rs17027006 | A | REC | 2.288 | (0.3662 | 14.3) | 0.3759 |
| 2 | rs12469506 | A | REC | 2.288 | (0.3662 | 14.3) | 0.3759 |
| 2 | rs10197862 | A | REC | 0.8676 | (0.4427 | 1.7) | 0.6792 |
| 2 | rs111533915 | C | REC | 0.6501 | (0.1266 | 3.337) | 0.6058 |
| 2 | rs148548829 | C | REC | 2.997 | (0.8009 | 11.21) | 0.103 |
| 2 | rs6751967 | A | REC | 0.7369 | (0.1388 | 3.912) | 0.72 |
| 2 | rs6704565 | G | REC | 2.288 | (0.3662 | 14.3) | 0.3759 |
| 2 | rs11123923 | A | REC | 0.6501 | (0.1266 | 3.337) | 0.6058 |
| 2 | rs4988956 | G | REC | 0.7369 | (0.1388 | 3.912) | 0.72 |
| 2 | rs4988956 | G | REC | 0.8676 | (0.4427 | 1.7) | 0.6792 |
| 2 | rs114130235 | A | REC | 1.141 | (0.4718 | 2.757) | 0.7702 |
| 2 | rs10192036 | A | REC | 0.7369 | (0.1388 | 3.912) | 0.72 |
| 2 | rs10192157 | A | REC | 1.141 | (0.4718 | 2.757) | 0.7702 |
| 2 | rs10206753 | G | REC | 2.997 | (0.8009 | 11.21) | 0.103 |
| ***IL33* gene** | | | | | | | |
| \| ***Aggregatibacter actinomycetemcomitans*** \| \| --- \| | | | | | | | |
| **CHR** | **SNV** | **Variant allele A1** | **Model** | **OR_Adjusted_ (95%CI)** | | | **p-value** |
| 9 | rs72614080 | A | ADD | 0.6504 | (0.2829 | 1.495) | 0.3111 |
| 9 | rs2066362 | A | ADD | 1.295 | (0.8369 | 2.005) | 0.2457 |
| 9 | rs1891385 | C | ADD | 0.6136 | (0.2909 | 1.294) | 0.1996 |
| 9 | rs118148121 | T | ADD | 2.856 | (0.7116 | 11.46) | 0.1388 |
| 9 | rs10435816 | G | ADD | 1.151 | (0.7902 | 1.678) | 0.4631 |
| 9 | rs12551256 | G | ADD | 0.8119 | (0.5376 | 1.226) | 0.3219 |
| 9 | rs7025417 | G | ADD | 0.8998 | (0.5726 | 1.414) | 0.6472 |
| 9 | rs78100995 | C | ADD | 0.8265 | (0.4119 | 1.658) | 0.5917 |
| 9 | rs1330383 | A | ADD | 1.185 | (0.7993 | 1.756) | 0.3988 |
| 9 | rs10975519 | A | ADD | 1.095 | (0.7384 | 1.622) | 0.6529 |
| 9 | rs142772030 | A | ADD | 6.232 | (0.6198 | 62.66) | 0.1202 |
| 9 | rs16924241 | G | ADD | 2.18 | (0.5623 | 8.448) | 0.2597 |
| 9 | rs1048274 | A | ADD | 1.081 | (0.7256 | 1.61) | 0.7026 |
| 9 | rs16924243 | G | ADD | 1.293 | (0.7228 | 2.313) | 0.3865 |
| 9 | rs72614080 | A | DOM | 0.6504 | (0.2829 | 1.495) | 0.3111 |
| 9 | rs2066362 | A | DOM | 1.568 | (0.9092 | 2.704) | 0.1057 |
| 9 | rs1891385 | C | DOM | 0.6187 | (0.2899 | 1.321) | 0.2147 |
| 9 | rs118148121 | T | DOM | 2.856 | (0.7116 | 11.46) | 0.1388 |
| 9 | rs10435816 | G | DOM | 1.158 | (0.6512 | 2.06) | 0.6171 |
| 9 | rs12551256 | G | DOM | 0.7339 | (0.4281 | 1.258) | 0.2605 |
| 9 | rs7025417 | G | DOM | 0.9795 | (0.5677 | 1.69) | 0.9407 |
| 9 | rs78100995 | C | DOM | 0.7591 | (0.3596 | 1.602) | 0.4695 |
| 9 | rs1330383 | A | DOM | 1.155 | (0.6731 | 1.98) | 0.6017 |
| 9 | rs10975519 | A | DOM | 1.006 | (0.5872 | 1.722) | 0.9836 |
| 9 | rs142772030 | A | DOM | 6.232 | (0.6198 | 62.66) | 0.1202 |
| 9 | rs16924241 | G | DOM | 2.18 | (0.5623 | 8.448) | 0.2597 |
| 9 | rs1048274 | A | DOM | 0.9522 | (0.5555 | 1.632) | 0.8586 |
| 9 | rs16924243 | G | DOM | 1.236 | (0.6529 | 2.341) | 0.5148 |
| 9 | rs72614080 | A | DOM | 0.6504 | (0.2829 | 1.495) | 0.3111 |
| 9 | rs2066362 | A | DOM | 1.568 | (0.9092 | 2.704) | 0.1057 |
| 9 | rs1891385 | C | DOM | 0.6187 | (0.2899 | 1.321) | 0.2147 |
| 9 | rs118148121 | T | DOM | 2.856 | (0.7116 | 11.46) | 0.1388 |
| 9 | rs10435816 | G | DOM | 1.158 | (0.6512 | 2.06) | 0.6171 |
| 9 | rs12551256 | G | DOM | 0.7339 | (0.4281 | 1.258) | 0.2605 |
| 9 | rs7025417 | G | DOM | 0.9795 | (0.5677 | 1.69) | 0.9407 |
| 9 | rs78100995 | C | DOM | 0.7591 | (0.3596 | 1.602) | 0.4695 |
| 9 | rs1330383 | A | DOM | 1.155 | (0.6731 | 1.98) | 0.6017 |
| 9 | rs10975519 | A | DOM | 1.006 | (0.5872 | 1.722) | 0.9836 |
| 9 | rs142772030 | A | DOM | 6.232 | (0.6198 | 62.66) | 0.1202 |
| 9 | rs16924241 | G | DOM | 2.18 | (0.5623 | 8.448) | 0.2597 |
| 9 | rs1048274 | A | DOM | 0.9522 | (0.5555 | 1.632) | 0.8586 |
| 9 | rs16924243 | G | DOM | 1.236 | (0.6529 | 2.341) | 0.5148 |
| 9 | rs118148121 | T | DOM | 2.856 | (0.7116 | 11.46) | 0.1388 |
| 9 | rs10435816 | G | DOM | 1.158 | (0.6512 | 2.06) | 0.6171 |
| 9 | rs12551256 | G | DOM | 0.7339 | (0.4281 | 1.258) | 0.2605 |
| 9 | rs7025417 | G | DOM | 0.9795 | (0.5677 | 1.69) | 0.9407 |
| 9 | rs78100995 | C | DOM | 0.7591 | (0.3596 | 1.602) | 0.4695 |
| 9 | rs1330383 | A | DOM | 1.155 | (0.6731 | 1.98) | 0.6017 |
| 9 | rs10975519 | A | DOM | 1.006 | (0.5872 | 1.722) | 0.9836 |
| 9 | rs142772030 | A | DOM | 6.232 | (0.6198 | 62.66) | 0.1202 |
| 9 | rs16924241 | G | DOM | 2.18 | (0.5623 | 8.448) | 0.2597 |
| 9 | rs1048274 | A | DOM | 0.9522 | (0.5555 | 1.632) | 0.8586 |
| 9 | rs16924243 | G | DOM | 1.236 | (0.6529 | 2.341) | 0.5148 |

CHR: Chromosome; OR_Adjusted_: odds ratio adjusted for total years of education, diagnosis of asthma, flossing, age, body mass index (BMI), mouth breathing habit, and PC1; 95%CI: confidence interval of 95%; p: statistical significance level (p≤0.05).
